# Oral microbiota related to allergy in Norwegian adults

**DOI:** 10.1016/j.jacig.2025.100435

**Published:** 2025-02-05

**Authors:** Mikyeong Lee, Hilde Kristin Vindenes, Farnaz Fouladi, Rajesh Shigdel, James M. Ward, Shayamal D. Peddada, Stephanie J. London, Randi Jacobsen Bertelsen

**Affiliations:** a Immunity Inflammation and Disease Laboratory, National Institute of Environmental Health Sciences, Research Triangle Park, NC; b Department of Occupational Medicine, Haukeland University Hospital, Bergen, Norway; c Department of Clinical Science, University of Bergen, Bergen, Norway; d Biostatistics & Computational Biology Branch, National Institute of Environmental Health Sciences, Research Triangle Park, NC; e Integrative Bioinformatics Support Group, National Institute of Environmental Health Sciences, Research Triangle Park, NC; f Oral Health Centre of Expertise in Western Norway–Vestland, Bergen, Norway

**Keywords:** Bacteria, allergy and immunology, host microbial interactions, microbiome, dysbiosis

## Abstract

**Background:**

Oral microbiome composition has been linked to onset and progression of several localized and systemic diseases. Associations with allergy in adults have been less explored.

**Objective:**

We sought to identify oral microbiota associated with allergy outcomes in adults using high-throughput sequencing data.

**Methods:**

We characterized bacterial communities of gingival samples from 453 Norwegian adults (average age, 28 years) using 16S rRNA gene amplicon sequencing. We examined more than 2200 bacterial taxa in relation to self-reported current asthma, eczema, or rhinitis, and seroatopy (IgE > 0.70 kU/L). We used linear regression to determine whether overall bacterial diversity differed by each allergic outcome and analysis of composition of microbiomes with bias correction (ANCOM-BC2) to identify differentially abundant taxa.

**Results:**

Less diverse oral bacterial communities were observed (*P* < .05) in individuals with atopy or rhinitis compared with those without. Bacterial diversity did not differ by asthma and eczema status. While no bacterial taxa were differentially abundant by asthma, many were differentially abundant (*P* < .05 after multiple-testing correction) in relation to atopy, eczema, and rhinitis. These taxa include several from the genera *Leptotrichia and Fusobacterium*. Some, including *Streptococcus*, were previously implicated in respiratory health, whereas others were novel. We also found taxa related to nasal medication use in individuals with rhinitis. Notably, microbial network interconnections differed by allergy status.

**Conclusions:**

Bacterial community compositions of oral gingival samples may play a role in allergic outcomes in adults. These findings could contribute to the development of novel treatment strategies.

Allergic disease is one of the most common chronic health conditions. It affects about 20% of the global population and is increasing in prevalence.^1^ Allergy negatively affects quality of life and is a major public health concern.^2^ Compared with genetic and other environmental factors involved in the etiology of allergic disease, little is known with respect to oral microbiome.

There is growing evidence supporting the contribution of oral hygiene to allergic diseases. In a large epidemiologic study, individuals reporting poor oral health were more likely to have asthma and allergic rhinitis diagnoses.^3^ Despite recent advances in high-throughput sequencing technology, findings based on thorough interrogation of oral microbes in relation to allergy are limited, with data predominantly from studies in childhood with moderate sample sizes.^4^, ^5^, ^6^ A study using 16S rRNA gene amplicon sequencing to examine oral wash samples from 66 adults identified microbiota related to atopy.^7^ To our knowledge, no large studies have examined associations between oral microbes and various allergic outcomes in adults using high-throughput sequencing methods.

We characterized bacterial community compositions using 16S rRNA gene amplicon sequencing in oral gingival samples from 453 adults enrolled in a community-based study in Bergen, Norway. We explored whether overall bacterial diversity and differential abundance of individual bacterial taxa differed by 4 allergic outcomes: asthma, atopy, eczema, and rhinitis. In addition, we interrogated network interconnections by the various allergy conditions to explore altered interplay among microbial communities.

## Methods

### Study population

Participants were enrolled in the community-based Respiratory Health in Northern Europe, Spain, and Australia study.^8^ There were 621 participants who completed questionnaires and clinical examination at a clinic visit (the Bergen study center) in the period 2014 to 2015. Of these, 468 adults (aged ≥18 years) had complete data on microbiota, allergic outcomes, and covariates. After excluding 14 participants reporting antibiotic use during the past 4 weeks and 1 without information on recent antibiotic use, 453 individuals were included in our association analyses. The study was approved by the Regional Committee for Medical and Health Research Ethics in Western Norway (approval no. 2012/1077). Written informed consent was obtained from all participants.

### Allergic outcomes

We analyzed 4 allergic outcomes: asthma, eczema, rhinitis, and atopy. We defined current asthma on the basis of questionnaire responses: “yes” to “Have you had an attack of asthma in the last 12 months?” or “yes” to “Are you currently taking any medicine (including inhalers, aerosols, or tablets) for asthma?” Current eczema was based on responses to 4 questions: (1) “Have you ever had eczema or any kind of skin allergy?”; (2) “How old were you when you first had eczema or skin allergy?”; (3) “Have you ever had an itchy rash that was coming and going for at least 6 months?”; and (4) “Have you had this itchy rash in the last 12 months?” Current rhinitis was based on responses to 2 questions: “Have you ever had a problem with sneezing or a runny or a blocked nose when you did not have a cold or the flu?” and “Have you had a problem with sneezing or a runny or a blocked nose when you did not have a cold or the flu in the last 12 months?” In individuals with rhinitis, we defined nasal medication use (never, last week, or past 12 months) on the basis of responses to 4 questions: (1) “Have you ever used any medication to treat nasal disorders?”; (2) “Have you used any of the following nasal sprays for the treatment of your nasal disorder?”; (3) “Have you used any of these nasal sprays in the last 12 months?”; and (4) “Have you used any of these nasal sprays in the last week?” Participants were shown a list of medications, including antihistamines and steroid nasal sprays. Current atopy was based on serum IgE levels more than 0.7 kU/L to at least 1 of 5 common allergens: cat, timothy grass, *Cladosporium*, birch, and house dust mite. Specific IgE levels were measured by ImmunoCAP (Phadia AB, Uppsala, Sweden) according to standardized laboratory methods at Haukeland University Hospital in Bergen, Norway. We also studied combined outcomes of atopy and other allergies: asthma and atopy (neither, atopy without asthma, asthma without atopy, and asthma with atopy), eczema and atopy (neither, atopy without eczema, eczema without atopy, and eczema with atopy), and rhinitis and atopy (neither, atopy without rhinitis, rhinitis without atopy, and rhinitis with atopy).

### Gingival sample collection, sequencing, and oral microbiota

Details on gingival sample collection and sequencing were previously described.^9^ In brief, subgingival fluid was collected from the gingival crevice at 5 sites using sterile paper points (PROTAPER, Jacobsen Dental, Bergen, Norway). The paper points were frozen (−80°C) directly after collection in 2-mL microtubes (Biopur Safe-Lock Tubes, Eppendorf SE, Hamburg, Germany) without buffer. Five paper points per individual were pooled for bacterial DNA extraction. The DNA library pool based on the 16S rRNA gene amplicons was sequenced with Illumina MiSeq platform (Illumina, Inc, San Diego, Calif) according to the manufacturer’s guidelines. DNA extraction and sequencing were performed at the Microbiome Core Facility of the University of North Carolina at Chapel Hill, NC. Samples were processed at 2 time points (batch 1 in 2016 and batch 2 in 2019) with the same procedure and by the same laboratory technician. Preprocessing of sequencing data was conducted using the Quantitative Insights Into Microbial Ecology (QIIME) software.^10^^,^^11^ Sequencing output from the Illumina MiSeq was converted to fastq format and demultiplexed using Illumina Bcl2Fastq 2.18.0.12. The resulting paired-end reads were processed using QIIME2 (2018.11 release). Index and linker primer sequences were trimmed using the QIIME2 invocation of cutadapt. The resulting paired-end reads were processed with DADA2 through QIIME2 including merging paired ends, quality filtering, error correction, and chimera detection.^12^ Amplicon sequencing variants (ASVs) from DADA2 were assigned to taxonomic identifiers using the q2-feature-classifier plugin^13^ against the expanded Human Oral Microbiome Database (version 15.01), a comprehensive database with well-curated 16S rRNA gene reference sequences from sites along the human aerodigestive tract.^14^

Of 29,043 ASVs identified from the 453 gingival samples, we excluded 26,785 having more than 0.005% of the total number of sequence reads (2,241) across all the samples.^15^ The resulting total of 2,258 ASVs (2,254 assigned to 10 phyla and 4 unassigned) were included in association analyses. Sequence reads per sample ranged from 8,669 to 240,758 with an average of 89,466 ± 41,590. Henceforth, we refer to an ASV as a bacterial taxon when appropriate.

### Associations between overall bacterial diversity and allergy outcomes

To determine overall bacterial diversity within each sample (alpha bacterial diversity), we calculated richness (representing the number of individual microbial taxon), Shannon index^16^ (reflecting both richness and the relative abundance of each taxon), and Faith phylogenetic diversity^17^ (PD; the sum of branch length of all taxa). Unlike the other 2 alpha diversity metrics, Faith PD takes phylogenetic relationships among taxa examined into account and thus differentiates a sample comprising highly related microorganisms (eg, all from the same family or genus) from a sample comprising microorganisms with greater phylogenetic distances (eg, organisms from different phyla or classes). To examine whether a bacterial diversity measure differs by each allergic outcome, we used linear regression with adjustment for age, sex, cigarette smoking (former or current, compared with never), body mass index (kg/m^2^), and batch (1 or 2).

To compare bacterial community compositions between samples (beta bacterial diversity) by allergic outcomes, we used the microbiome regression-based kernel association test (MiRKAT).^18^ MiRKAT models the log-odds of the outcome using a mixed model and tests whether the variance component attributable to pairwise distance (or dissimilarity) among the samples’ bacterial communities is 0. We calculated pairwise distances using the unique fraction metric (UniFrac)^19^ considering phylogenetic distance among taxa in a phylogenetic tree. Additional weighting was given to take abundances into account (weighted UniFrac). We quantified compositional dissimilarity between samples by comparing abundances using the Bray-Curtis dissimilarity metric.^20^

To avoid any bias due to varying sequencing depth among samples in diversity analyses, the ASV abundance data were rarefied to the minimum number of sequences (8669) across samples. We used R (version 4.2.1; R Project for Statistical Computing) to summarize characteristics of the study population and perform association analyses of the alpha diversity measures. We calculated alpha diversity measures using the function alpha() in the R package microbiome (version 1.18.0).^21^ Faith PD was calculated using the pd() function in the R package picante (version 1.8.2).^22^ For the beta diversity analysis, we used the R package MiRKAT (version 1.2.3).^18^ We set a threshold of *P* value less than .05 for statistical significance for the diversity analyses.

### Associations between individual bacterial taxa and allergy outcomes

To determine whether specific bacterial taxa were differentially abundant in relation to allergic outcomes, we implemented analysis of compositions of microbiomes with bias correction (ANCOM-BC2).^23^^,^^24^ The original ANCOM models the log-ratios of bacterial abundances in each sample with a linear model and accommodates dependencies and correlations among the relative abundances of the taxa.^25^ The updated ANCOM-BC2 estimates and corrects sample-specific (sampling fraction) as well as taxon-specific (sequencing efficiency) biases.^23^^,^^24^ For multilevel combined allergy outcomes, we performed the Dunnett test,^26^ which executes multiple pairwise comparisons against a prespecified group (individuals without the outcome of interest). The multiple pairwise directional test in ANCOM-BC2 gives better power by controlling the mixed directional false-discovery rate, which is the combination of false-discovery rate related to multiple testing, multiple pairwise comparisons, and directional tests within each pairwise comparison.^24^^,^^27^ In our example, each of the 3 groups (atopy without rhinitis, rhinitis without atopy, and rhinitis with atopy) was compared with a group without the 2 outcomes (neither) when we analyzed the combined outcome of rhinitis and atopy. We used unrarefied microbiome data because the use of log-ratios in ANCOM accounts for variation in sequencing depth across samples. To enable log-transformation of the abundance count data, which include many zero counts by nature, one typically adds a positive real number called pseudo-count. For some taxa, depending on the pseudo-count, the statistical test may result in a false positive, that is, an inflated false-discovery rate. To deal with this, ANCOM-BC2 determines whether a taxon is sensitive to the choice of pseudo-count and filters all such taxa as potentially false positives. In our analysis, we implemented this feature with the default cutoff value of 1.^24^ We adjusted for the aforementioned covariates using the ancombc2() function in the updated ANCOM-BC R package (version 2.4.0) and R (version 4.3.1). To correct for multiple testing, we applied the Holm-Bonferroni procedure^28^ at 5%, which is suitable for handling arbitrary dependence structure among the *P* values obtained from differential abundance analysis.^24^

### Microbial network construction and visualization

Spearman rank-order correlations were assessed at the genus level using Sparse Estimation of Correlations among Microbiomes (SECOM).^29^ SECOM corrects for sample- and taxon-specific biases and accounts for the compositionality of microbiome data. To avoid any inflated correlation coefficients due to small variances and to account for near singularity and multicollinearity of microbiome data, we regularized small eigenvalues of the covariance matrix using winsorization.^30^ Spearman correlation estimates were calculated using the secom_linear() function, with default options, in the ANCOM-BC R package (version 2.4.0). This correlation analysis included genera present in more than 50% of all samples. Microbial networks were constructed on the basis of the Spearman correlations (>0.5) obtained from SECOM and visualized using the Network Construction and comparison for Microbiome data R package.^31^

## Results

In this Norwegian population, the average age was 28 years (range, 18-47 years). As expected, rhinitis (45%) and atopy (37%) were more common than current asthma (6%) and eczema (12%) (Table I). When we explored outcomes combined with atopy, rhinitis with atopy (24%) was more common than other combined outcomes. The population was generally of normal body mass index (mean ± SD, 25 ± 5).

**Table I:** Characteristics of study participants (N = 453)

Characteristics	N (%)∗ or mean ± SD
Age (y)	28 ± 7
Sex	
Female	213 (47)
Male	240 (53)
Cigarette-smoking status	
Never	315 (70)
Former	74 (16)
Current	64 (14)
Body mass index (kg/m^2^)	25 ± 5
Current asthma	
No	427 (94)
Yes	26 (6)
Current atopy	
No	284 (63)
Yes	169 (37)
Current eczema	
No	398 (88)
Yes	55 (12)
Current rhinitis	
No	250 (55)
Yes	203 (45)
Current rhinitis (recent use of nasal medications)†	
No medication use	87 (49)
Medication use in the past 12 mo (not past week)	59 (33)
Medication use in the last week	32 (18)
Current asthma combined with atopy	
Neither	273 (60)
Atopy without asthma	154 (34)
Asthma without atopy	11 (2)
Asthma with atopy	15 (3)
Current eczema combined with atopy	
Neither	253 (56)
Atopy without eczema	145 (32)
Eczema without atopy	31 (7)
Eczema with atopy	24 (5)
Current rhinitis combined with atopy	
Neither	190 (42)
Atopy without rhinitis	60 (13)
Rhinitis without atopy	94 (21)
Rhinitis with atopy	109 (24)
Batch	
2016	269 (59)
2019	184 (41)

∗ Percentages may not add to exactly 100 because of rounding.

† After excluding individuals with current rhinitis without information on nasal medication use, 178 were included in the rhinitis and medication use analysis.

**Fig 1:**
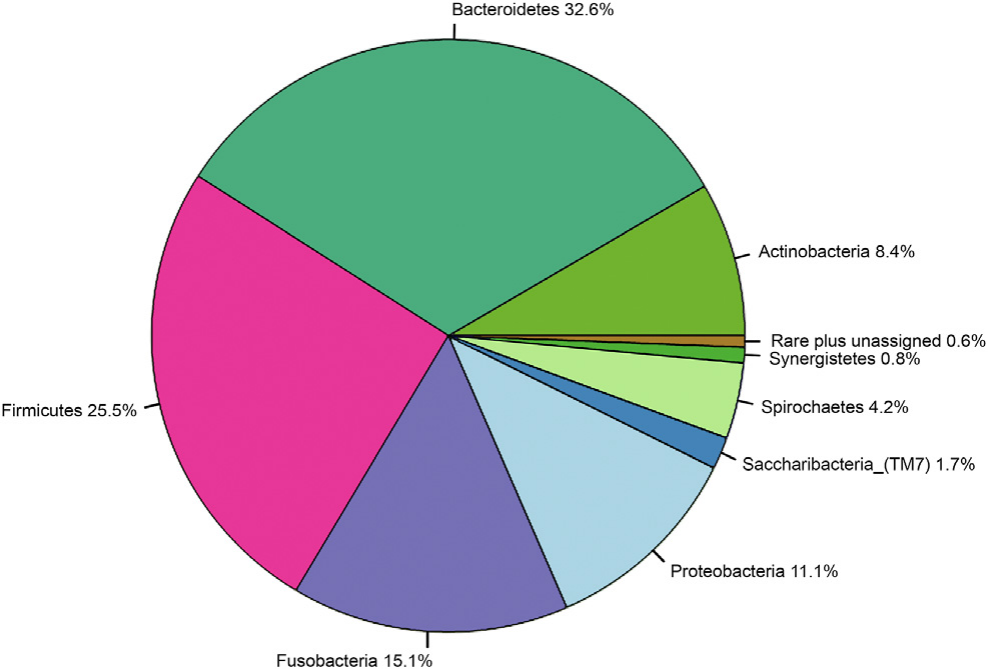
Proportion of ASVs at the phylum level. A total of 2254 ASVs assigned to 10 phyla and 4 unassigned. The “rare plus unassigned” category includes 8 ASVs (0.4%) from the phylum Absconditabacteria_(SR1), 2 ASVs (0.1%) from the phylum Gracilibacteria_(GN02), and 4 unassigned (0.2%).

**Fig 2:**
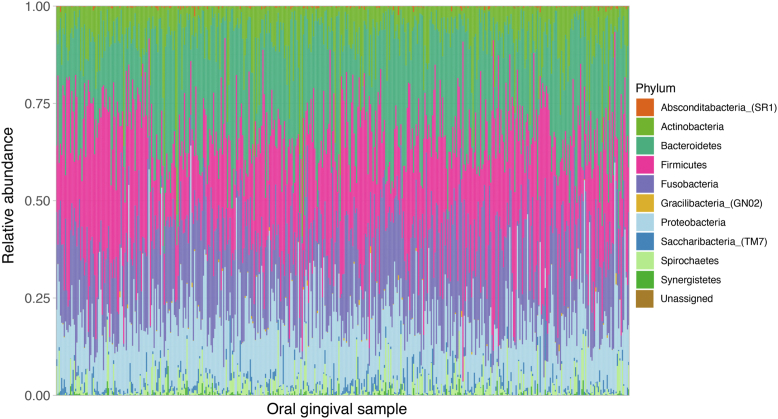
Relative abundance at the phylum level across all gingival samples (N = 453). This figure shows the phylum level summary of relative abundance in each sample. The x-axis indicates gingival samples examined, and the y-axis represents relative abundance at the phylum level.

Our oral microbiome data included 2254 bacterial taxa from 10 phyla and 4 taxa unassigned. Predominant phyla, on the basis of numbers of bacterial taxa assigned, were Bacteroidetes (33%), Firmicutes (26%), Fusobacteria (15%), and Proteobacteria (11%) (Fig 1). Bacterial community compositions at the phylum level varied by sample (Fig 2).

**Fig 3:**
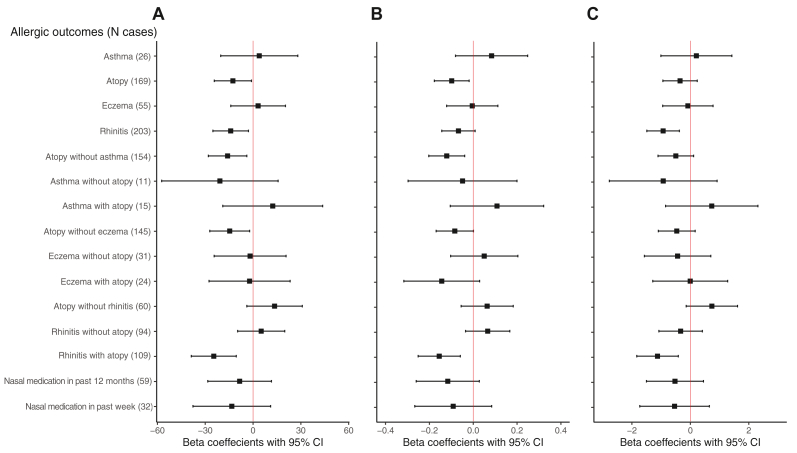
Associations between overall bacterial diversity (alpha diversity) and allergic outcomes. This figure summarizes linear regression association results between alpha diversity measures and allergic outcomes. Included covariates were age, sex, smoking (never/former/current), body mass index, and batch (1/2). For each individual allergic outcome (asthma, atopy, eczema, and rhinitis), bacterial diversity measures in individuals with each outcome of interest were compared with those without that outcome (N = 453). For combined allergic outcomes (asthma with atopy, eczema with atopy, and rhinitis with atopy), individuals of each subgroup were compared with those having neither atopy nor the other outcome under consideration (N = 453). For example, individuals with atopy without asthma, asthma without atopy, or asthma with atopy were compared with those with neither asthma nor atopy. Individuals with nasal medication use in the past 12 months or in the past week were compared with individuals with rhinitis reporting no use of nasal medications (N = 178). **A,** Richness. **B,** Shannon index. **C**, Faith PD.

When we compared overall bacterial diversity within each sample (alpha bacterial diversity) by allergic outcomes, associations slightly varied among the 4 allergic outcomes. No significant differences in overall bacterial diversity were observed in individuals with asthma or eczema (Fig 3; see also Table E1 in this article’s Online Repository at www.jaci-global.org). However, richness (the number of bacterial taxa observed) and Shannon index (reflecting the number of bacterial taxa along with abundances of individual bacterial taxa) were significantly lower in individuals with atopy than in those without atopy (*P* < .05). When we accounted for the phylogenetic distances among taxa examined (Faith PD), individuals with atopy had lower Faith PD but the association was not significant (*P* = .24). Both richness and Faith PD values were significantly lower in individuals with rhinitis than in those without rhinitis (*P* < .05). Shannon index was also lower in individuals with rhinitis, but the association was not statistically significant (*P* = .08).

When we examined asthma combined with atopy, individuals with atopy alone exhibited lower bacterial diversity compared with individuals with neither atopy nor asthma (Fig 3; Table E1). This pattern was also observed for the combined eczema and atopy outcome. When we interrogated rhinitis together with atopy, significantly less diverse bacterial communities were observed only in individuals with both rhinitis and atopy compared with neither (*P* < .05 for all 3 diversity measures).

When we compared bacterial community compositions between samples (beta bacterial diversity), we found no significant differences in distances or dissimilarities for asthma, atopy, and eczema. However, the phylogenetic distance among taxa identified in individuals with rhinitis differed from the distance in individuals without rhinitis (*P* = .02 for unweighted UniFrac, without taking taxa abundances into account) (see Table E2 in this article’s Online Repository at www.jaci-global.org).

**Fig 4:**
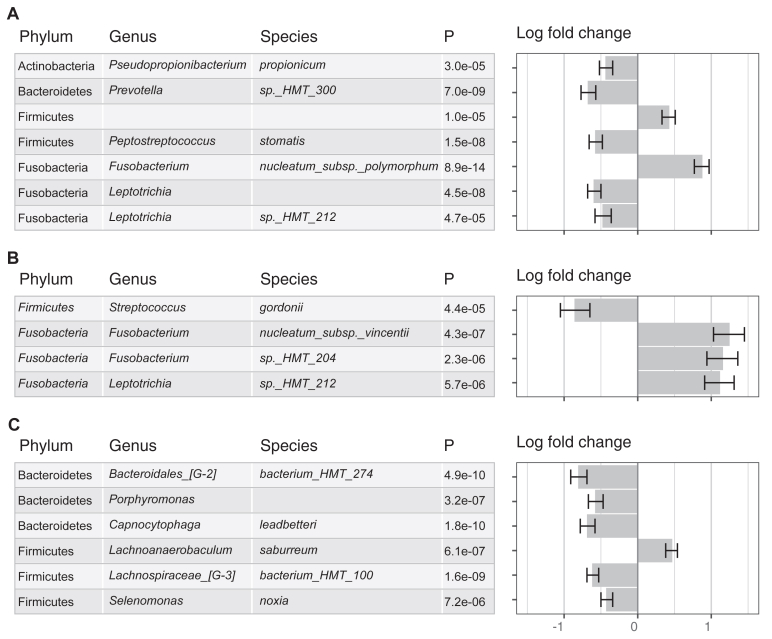
Taxa significantly differentially abundant in relation to atopy, eczema, or rhinitis. On the left are taxonomic classifications (phylum, genus, and species) and *P* values for each taxon. On the right are the estimated mean differences of absolute abundance between groups on the natural log scale. The taxa graphed showed differential abundance (*P* < .05 after multiple-testing correction) in relation to an allergic outcome after adjusting for age, sex, smoking (never/former/current), body mass index, and batch (1/2) (N = 453). **A,** Atopy. **B**, Eczema. **C,** Rhinitis.

In analyses of individual bacterial taxa, we found 17 distinct taxa exhibiting significant differential abundance by atopy, eczema, or rhinitis (*P* < .05 after multiple-testing correction) (Fig 4; see also Table E3 in this article’s Online Repository at www.jaci-global.org). Of the 17 taxa, 2 were from the same species: *Leptotrichia HMT_212* (phylum Fusobacteria). Seven taxa showed differential abundance by atopy. These include 3 from phylum Fusobacteria, including 2 (genus *Leptotrichia*) less abundant in individuals with atopy compared with those without atopy. Four taxa were associated with eczema. Of these, 3 were from phylum Fusobacteria (2 from genus *Fusobacterium* and 1 from genus *Leptotrichia*). All 3 were more abundant in individuals with eczema than in those without eczema. In addition, the species *Streptococcus gordonii* (phylum Firmicutes) was less abundant in individuals with eczema. For rhinitis, 6 taxa were differentially abundant (3 from phylum Bacteroidetes and 3 from phylum Firmicutes). Among these, the species *Lachnoanaerobaculum saburreum* (phylum Firmicutes) was more abundant in individuals with rhinitis, whereas 5 were less abundant in individuals with rhinitis.

Power is much more limited for asthma than for the other outcomes with only 26 cases. Although no taxon was associated with asthma after multiple-testing correction and an additional filtering step based on sensitivity analysis results, some taxa related to eczema or rhinitis were differentially abundant in relation to asthma (see Table E4 in this article’s Online Repository at www.jaci-global.org) with consistent directions of associations. One taxon (phylum Fusobacteria; genus *Fusobacterium*) that was significantly positively associated with eczema was also more abundant in individuals with asthma. A taxon (phylum Bacteroidetes; genus *Bacteroidales [G-2]*) negatively related to rhinitis showed lower abundance in individuals with asthma.

Some microbes were significantly related to multiple allergic outcomes. After multiple-testing correction, the species *Leptotrichia HMT_212* (phylum Fusobacteria) was related to atopy and also to rhinitis (Table E3). Several different taxa from the genus *Streptococcus* (phylum Firmicutes) were associated with eczema and other combined outcomes with atopy (see Tables E5-E7 in this article’s Online Repository at www.jaci-global.org). We also found microbes linked to a specific outcome. A taxon from species *Pseudopropionibacterium propionicum* (phylum Actinobacteria) was associated with atopy, but no taxon from the same phylum was related to other allergic outcomes (Table E3).

Examination of allergic outcomes combined with seroatopy revealed taxa not identified when we examined the outcomes separately. When we stratified individuals with asthma by atopy status, we found 5 taxa related to specific subtypes: 3 for asthma without atopy and 2 for asthma with atopy, each compared with neither (Table E5). When we analyzed eczema stratified by atopy, we found 4 taxa related to eczema without atopy and 2 related to eczema with atopy (Table E6). There were 6 taxa related to rhinitis without atopy, 4 related to rhinitis with atopy, and 1 (phylum Firmicutes; genus *Lachnospiraceae [G-3]*; species *bacterium HMT 100*) related to rhinitis with or without atopy (Table E7). Notably, several different taxa from genus *Prevotella* (phylum Bacteroidetes) were related to eczema without atopy and rhinitis without atopy.

In addition, we identified bacterial taxa associated with nasal medication use in individuals with rhinitis (*P* < .05 after multiple-testing correction). There were 11 taxa differentially abundant in relation to nasal medication use in the past 12 months compared with individuals with rhinitis but no medication use (see Table E8 in this article’s Online Repository at www.jaci-global.org). In addition, 4 taxa showed differential abundance in relation to use of nasal medications in the past week compared with individuals with rhinitis but without nasal medication use. In individuals with rhinitis, nasal medication use was positively related to oral microbes including taxa from genus *Provotella* (phylum Bacteroidetes). Some taxa from genus *Streptococcus* (phylum Firmicutes) and genus *Saccharibacteria (TM7) [G-3]* (phylum Saccharibacteria (TM7)) inversely related to medication use.

**Fig 5:**
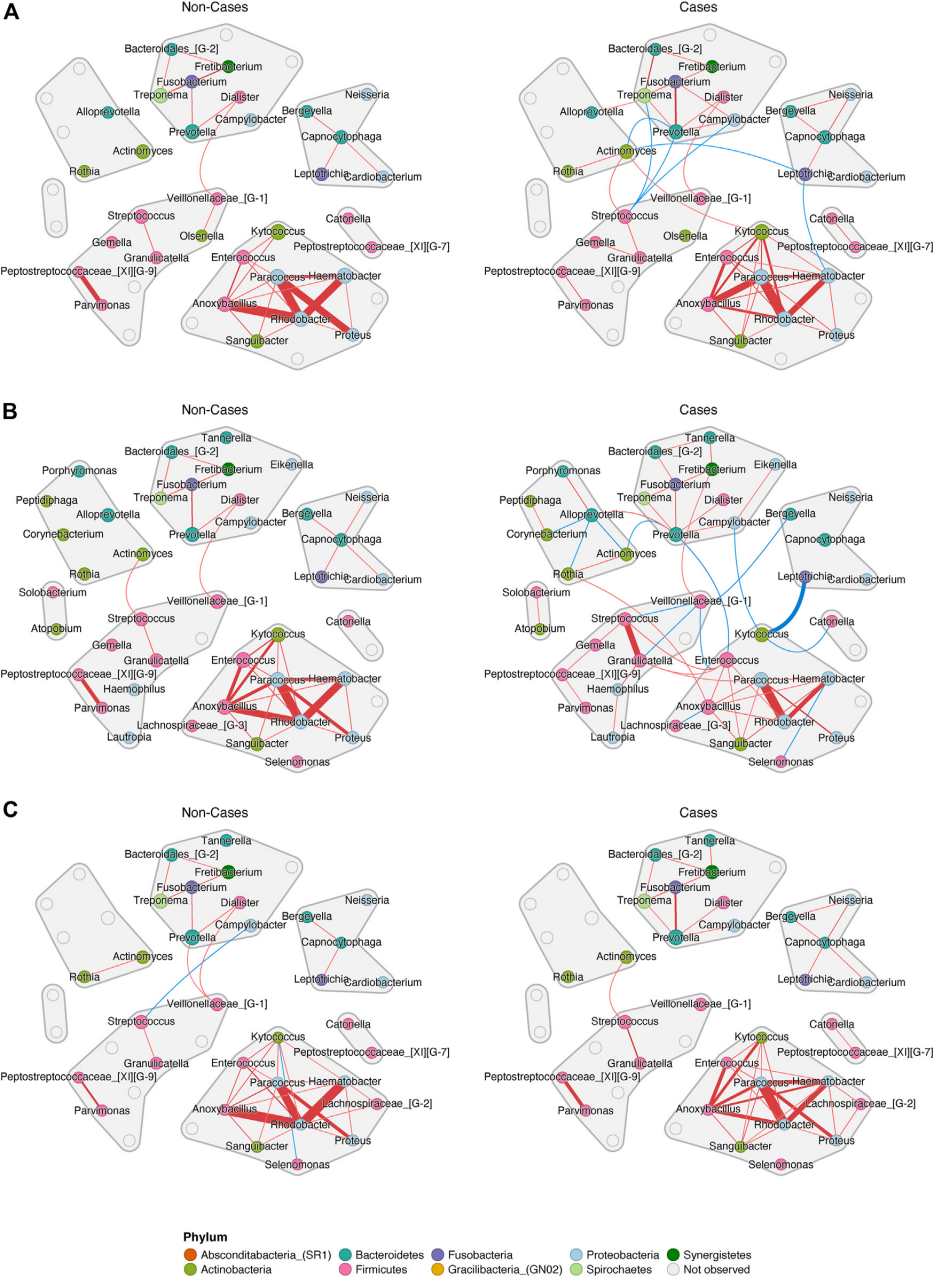
Microbial networks by allergy status. Spearman correlation–based networks were constructed separately in individuals with allergy (cases) and those without (non-cases). Graphs use a shared network layout. Nodes representing observed genus are colored by phylum. *Gray* nodes indicate genera not observed in that condition. Edges, showing absolute correlation coefficients greater than 0.5 and *P* values less than .001, are colored *red* for positive correlation and *blue* for negative correlation. Edge widths (weights) are based on the strength of associations. *Gray* border and shading indicate network clustering as determined using the fast greedy algorithm (https://doi.org/10.48550/arXiv.cond-mat/0408187). **A**, Atopy. **B,** Eczema. **C,** Rhinitis.

Examination of microbial networks revealed distinct microbial interactions by allergy status. Some genera were interconnected (Spearman correlations > 0.5) in individuals without allergy, but not in individuals with allergy, or vice versa. For example, the genus *Actinomyces* interacted with *Kytococcus* (both from phylum Actinobacteria) in individuals with atopy but not in individuals without atopy (Fig 5; see also Fig E1 in this article’s Online Repository at www.jaci-global.org). The genus *Leptotrichia* (phylum Fusobacteria) was linked to multiple genera from different phyla in individuals with atopy, but its connection to *Haematobacter* (phylum Proteobacteria) and *Actinomyces* (phylum Actinobacteria) was not present in individuals without atopy.

Studies have reported sex differences in the oral micobiome.^32^ The numbers of individuals with allergy were similar between male and female participants (see Table E9 in this article’s Online Repository at www.jaci-global.org), and directions of associations with overall bacterial diversity were consistent with ones from all participants together (see Table E10 in this article’s Online Repository at www.jaci-global.org). Some associations were significant in male participants, but not in female participants, and there was no evidence of interactions by sex. When conducting differential abundance analyses by sex, we observed 1 taxon (phylum Firmicutes; *Peptostreptococcaceae_[XI][G-4] bacterium_HMT_369*; log fold change, −2.8; *P* = 1.4 × 10^−6^) associated with asthma in female participants only, whereas no taxon was related to asthma when we analyzed all participants together. Many taxa were associated with atopy, eczema, or rhinitis in male- and female-specific results (see Tables E11 and E12 in this article’s Online Repository at www.jaci-global.org). Most genera identified in sex-stratified analyses overlapped with findings in all participants. Notably, some taxa were unique to sex-specific results of rhinitis, including the genera *Corynebacterium* (phylum Actinobacteria) in male participants and *Bergeyella* (phylum Bacteroidetes) in female participants.

### Conclusion

In this large study examining comprehensive bacterial profiles generated by high-throughput sequencing, we found significant associations between oral bacterial community compositions and allergic outcomes in adults. Less diverse oral bacterial communities were observed in individuals with atopy or rhinitis. We found many specific bacterial taxa differentially abundant in relation to atopy, eczema, or rhinitis. Notably, we observed altered microbial interplay in individuals with allergy compared with those without allergy. Among individuals with rhinitis, we also found taxa related to use of nasal medications. Our findings suggest a potential link between oral microbiota and allergy outcomes in adults. Identifying specific oral microbes related to allergies could help uncover underlying mechanisms and inform development of treatment strategies.

Oral bacterial diversity differed by some allergic outcomes but not others. Overall bacterial diversity was inversely associated with atopy and rhinitis, whereas no significant differences were observed by asthma and eczema. This is in line with previous findings of less diverse bacterial communities in the oral cavity in relation to atopy and asthma in adults^7^ and in children.^33^ Our study extends knowledge of oral bacterial diversity and allergy by increasing sample size and examining various allergy outcomes as well as use of commonly used nasal medications.

Microbes identified in relation to allergies in our data include ones reported in previous human microbiome studies. The genus *Streptococcus*, related to allergic outcomes in our study, was associated with asthma in an adult oral microbiome study (N = 66)^7^ and neutrophilic asthma, a more severe and persistent form of asthma, in an adult airway microbiome study (N = 167).^34^ The genus was also related to asthma control in an airway microbiome study of 319 asthmatic children.^35^ In our study, the genus *Capnocytophaga* was related to rhinitis and eczema and it has been previously related to asthma in children.^33^^,^^36^ A taxon from the genus *Selenomonas* was inversely related to rhinitis in our data. In previous studies, this genus was reported to be more abundant in healthy children than in children developing allergies during the first 7 years of life^37^ and to be inversely associated with asthma exacerbations despite the use of inhaled corticosteroids in saliva samples.^38^ Given that the oral microbiome composition changes from childhood to adulthood even in healthy individuals and different factors shape oral microbiome in children compared with adults,^39^^,^^40^ validating findings from previous studies of children in our adult data highlight contributions of specific oral microbiota to allergy across the life course. Investigation of how the progression of the oral microbiome from childhood to adulthood influences an allergic disease would be a useful topic for future research. The genus *Prevotella*, a bacterium previously related to several inflammatory conditions,^41^ showed associations with atopy, eczema, and rhinitis in our data. The genus *Treponema* was related to atopy and asthma in our data. In a study of airway microbiome and severe asthma (N = 40), the genus *Treponema* was related to obesity (body mass index ≥ 30 kg/m^2^) in individuals with asthma.^42^ This provides partial validation of our findings in the microbiome of other body sites known to be interconnected to oral microbiome. Our findings hint at specific oral microbes contributing to mechanisms shared across different allergic outcomes and specific characteristics in individuals with allergies. This is notable because the oral microbiome is the primary source of the lung microbiome and plays a key role in inflammation in lung and systemic health.^40^^,^^43^^,^^44^ Future studies of how the microbiome from various body sites differs in relation to allergic outcomes could provide valuable insights into underlying mechanisms.

By exploring microbial networks by allergy status, we were able to identify altered network interconnections in adults with allergy compared with those without allergy. There were some microbial interconnections observed among specific genera only in individuals with allergy or only in those without allergy. Some were linked to other genera more strongly in individuals with allergy than in those without, or vice versa. These network analysis results hint at the altered interplay of oral microbes in individuals with allergy.

Some oral bacteria associated with allergies in our data have previously been reported as constituents of the core oral microbiome in healthy adults.^45^ These include the genera *Streptococcus* (phylum Firmicutes), *Prevotella* (phylum Bacteroidetes), *Capnocytophaga* (phylum Bacteroidetes), and *Fusobacterium* (phylum Fusobacteria). This suggests that dysbiosis in core microbiomes plays a role in allergic conditions.

Our study has several limitations. The samples were collected only at a single time point. Thus, we are assuming that a sample reflects the usual oral microbial condition. However, this limitation would tend to bias our results toward the null rather than explain observed associations. Our investigation is cross-sectional, which makes it difficult to disentangle the directionality of the association: whether the oral microbiota contribute to allergy in adults or whether an atopic or allergic condition and medication use lead to changes in the oral microbiota. Unfortunately, we lack information on severity of allergy outcomes, which could have improved clinical implications of our study. However, in individuals with rhinitis, nasal medication use provided an index of currently active symptoms. Because of the limited number of individuals with rhinitis reporting medication use, this study lacks power to explore associations with different nasal medication types: antihistamines and steroids (see Table E13 in this article’s Online Repository at www.jaci-global.org). Participants had a limited age range (mean ± SD, 28 ± 7) and, consistent with the study being in Norway, a limited body mass index range (25 ± 5). Thus, this study is not well suited to the examination of effect modification by these factors. In addition, microbiome sequencing of the gingival samples was processed in 2 batches at different time points. However, the batch effect was taken into account by adjusting for the batch variable in statistical models. Although we are unable to quantify absolute levels of oral bacteria, we are able to quantify relative abundances of bacterial taxa and assess directions of associations.

One of the strengths of this study is the large sample size compared with previous oral microbiome studies in adults using 16S rRNA amplicon sequencing. Oral microbiome profiles differ by sample collection site, and oral microbes from gingival samples, unlike those collected from other sites, could reflect both local and systemic inflammation.^46^^,^^47^ Our results could be microbial signatures unique to gingival samples or common in microbes across different oral sites. Our definition of current atopy was objective on the basis of specific IgE with a strict threshold of 0.7 kU/L. We identified bacteria related to nasal medication use in individuals with rhinitis, which has been less explored in literature. Implementing high-throughput sequencing allowed us to overcome a major limitation of conventional culture-dependent methods and interrogate comprehensive bacterial profiles, including ones that are not culturable. Implementing the updated ANCOM-BC2 method^23^^,^^24^ enabled bias correction related to sample fraction and sequencing efficiency as well as evaluation of association results after sensitivity analysis for pseudo-count addition. Lastly, exploring microbial networks enabled identification of altered interplay of microbial communities by allergy status, which cannot be captured by analysis of individual bacterial taxa.

To our knowledge, this is the first large study of oral microbiome in relation to adult allergic outcomes in a general population. Compared with individuals without allergies, individuals with allergic outcomes had less diverse oral bacterial communities. We observed bacterial communities in oral gingival samples significantly associated with allergic outcomes in adults. We identified specific bacteria associated with certain allergic conditions, some in higher and some in lower abundance. Microbial networks in the oral cavity differed by allergy status. Modification of the oral microbiome has been proposed as strategy in control of chronic diseases including cancers, Alzheimer disease, and cardiovascular conditions.^48^ Our results from this comprehensive investigation suggest that such strategies could be relevant to allergic diseases.

## Disclosure statement

This project was funded by the Research Council of Norway (grant nos. 230827 and 273838) and the Western Norwegian Regional Health Authorities (grant no. 912128). The Respiratory Health in Northern Europe, Spain, and Australia study at the Bergen center was funded by the Research Council of Norway (grant nos. 214123 and 228174) and the Western Norwegian Regional Health Authorities (grant nos. 912011, 911892, and 911631). This project has received funding from the 10.13039/501100000781European Research Council under the European Union’s Horizon 2020 research and innovation program (grant agreement no. 804199). The research work of S.D.P. was supported (in part) by funding from the 10.13039/100030692Intramural Research Program of the 10.13039/100009633Eunice Kennedy Shriver National Institute of Child Health and Human Development, the National Institutes of Health, and the 10.13039/100000066National Institute of Environmental Health Sciences (NIEHS) intramural program (grant no. ZIA ES103390-01). The research work of F.F. was also funded (in part) by the NIEHS intramural program (grant no. ZIA ES103390-01). This work was supported by the Intramural Research Program of the National Institutes of Health, NIEHS (grant no. ZO1 ES43012).

Disclosure of potential conflict of interest: The authors declare that they have no relevant conflicts of interest.
