# Supplementary material for: Oral microbiota related to allergy in Norwegian adults
Source: J Allergy Clin Immunol Glob. 2025 Feb 5;4(2):100435. doi: 10.1016/j.jacig.2025.100435 (PMC11914992; doi:10.1016/j.jacig.2025.100435)
Supplement: Supplementary Figs and Tables [file mmc1.pdf]

Online Supplement.

Figure S1. Spearman correlation by allergy status.
Spearman correlation coefficients used for network visualization in Figure 5 are shown as heatmaps, using red for positive and blue for negative correlations. Genus level absolute correlations that exceed 0.5 with P-value < 0.001 are marked “X” and were defined as network edges in Figure 5. Rows and columns are organized by Genus and are split by Phylum.

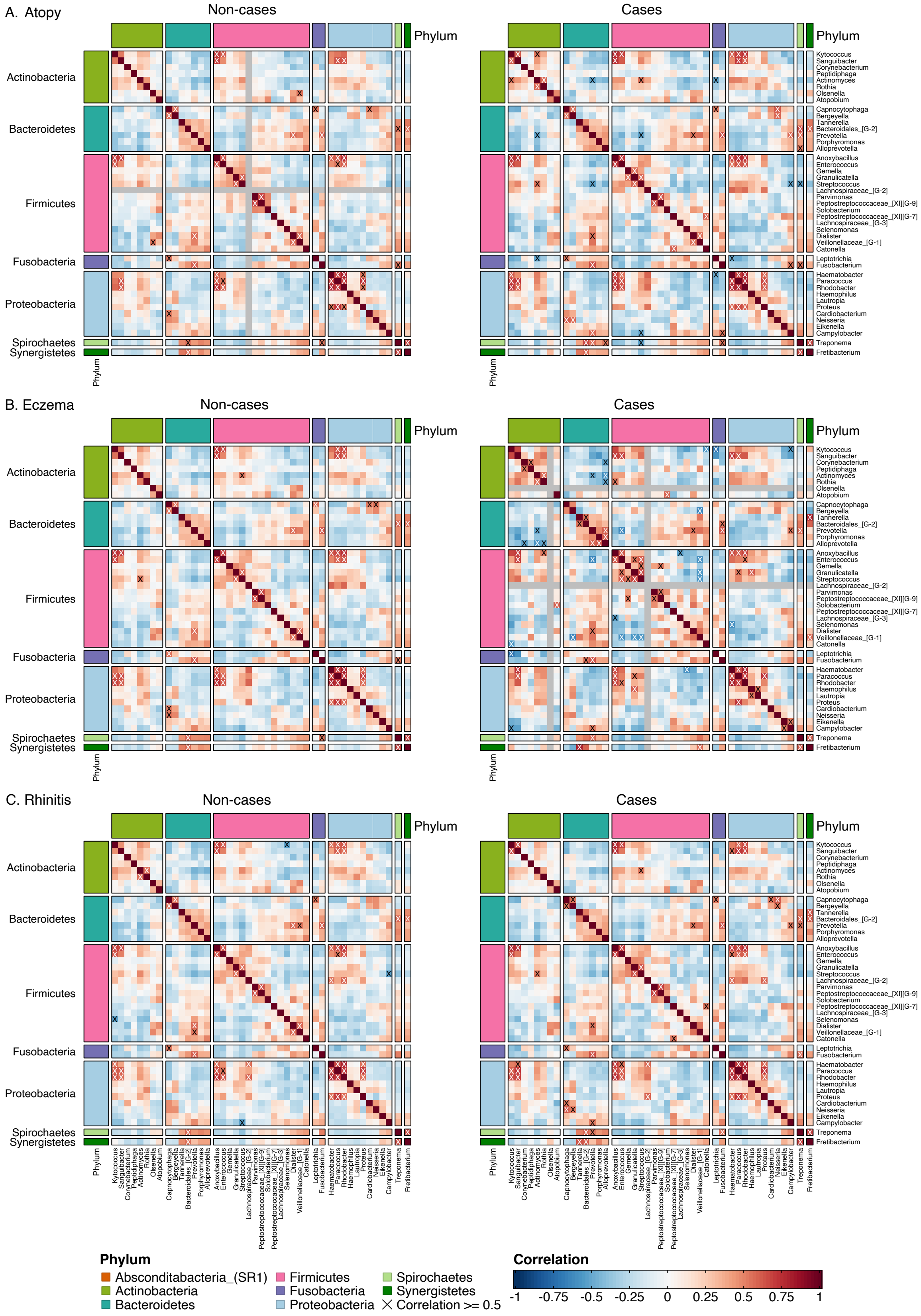

Supplemental Tables.

Table S1. Associations between overall bacterial diversity and allergy outcomes (N=453).

	Richness			Shannon Index			Faith's Phylogenetic Diversity		
	Coef	SE	P	Coef	SE	P	Coef	SE	P
Asthma	3.73	11.82	0.7523	0.08	0.08	0.3194	0.20	0.59	0.7400
Atopy	-12.11	5.70	0.0342	-0.09	0.04	0.0157	-0.34	0.29	0.2408
Eczema	3.04	8.39	0.7171	-0.004	0.06	0.9395	-0.08	0.42	0.8467
Rhinitis	-13.44	5.45	0.0141	-0.06	0.04	0.0832	-0.89	0.27	0.0011
Asthma and atopy combined									
Atopy without asthma	-15.31	5.90	0.0097	-0.12	0.04	0.0041	-0.48	0.30	0.1095
Asthma without atopy	-19.92	17.86	0.2652	-0.05	0.12	0.7011	-0.89	0.90	0.3241
Asthma with atopy	11.82	15.31	0.4404	0.10	0.10	0.3195	0.70	0.77	0.3647
Atopy and eczema combined									
Atopy without eczema	-14.03	6.12	0.0223	-0.08	0.04	0.0531	-0.45	0.31	0.1487
Eczema without atopy	-1.77	11.02	0.8728	0.05	0.07	0.5224	-0.42	0.56	0.4514
Eczema with atopy	-2.09	12.43	0.8667	-0.14	0.08	0.1040	-0.005	0.63	0.9940
Atopy and rhinitis combined									
Atopy without rhinitis	12.93	8.51	0.1294	0.06	0.06	0.2955	0.70	0.43	0.1012
Rhinitis without atopy	4.90	7.22	0.4976	0.06	0.05	0.2005	-0.32	0.36	0.3812
Rhinitis with atopy	-23.59	6.90	0.0007	-0.15	0.05	0.0017	-1.08	0.35	0.0021
Nasal medication use in individuals with rhinitis [a]									
Medication use in the past 12 months (not past week)	-8.06	9.76	0.4103	-0.11	0.07	0.1153	-0.50	0.48	0.2918
Medication use in last week	-12.77	11.90	0.2850	-0.09	0.09	0.3090	-0.52	0.58	0.3749

Adjusted for age, sex, smoking (never/former/current), body mass index, and batch (1/2).

[a] After excluding individuals with rhinitis without information on nasal medication use, 178 were used in the rhinitis and medication use analysis.

Table S2. P-values from the beta bacterial diversity analysis evaluating associations of bacterial community compositions between samples by allergy outcomes (N=453).

	Distance/dissimilarity metrics			
	Weighted UniFrac	Unweighted UniFrac	Bray-Curtis dissimilarity	Omnibus[a]
Asthma	0.8154	0.8107	0.8261	0.9820
Atopy	0.3298	0.1549	0.7734	0.3750
Eczema	0.7426	0.8001	0.5733	0.8750
Rhinitis	0.6071	0.0221	0.0839	0.0735

Adjusted for age, sex, smoking (never/former/current), body mass index, and batch (1/2).
[a]Omnibus p-value considering all three kernels from the three metrics: weighted/unweighted UniFrac distance and Bray-Curtis dissimilarity.

Table S3. Bacterial taxa differentially abundant in relation to atopy, eczema, and rhinitis (N=453).

	Atopy					Eczema					Rhinitis				
Taxon	LFC	SE	P	Q	S	LFC	SE	P	Q	S	LFC	SE	P	Q	S
Seven taxa associated with atopy															
p__Actinobacteria; c__Actinobacteria; o__Propionibacteriales; f__Propionibacteriaceae; g__Pseudopropionibacterium; s__propionicum	-0.43	0.09	3.0E-05	0.0204	T	-0.51	0.20	0.0142	1.0000	T	-0.16	0.08	0.0589	1.0000	F
p__Bacteroidetes; c__Bacteroidia; o__Bacteroidales; f__Prevotellaceae; g__Prevotella; s__sp._HMT_300	-0.67	0.10	7.0E-09	5.4E-06	T	-0.04	0.21	0.8403	1.0000	T	-0.07	0.09	0.4453	1.0000	T
p__Firmicutes	0.42	0.09	1.0E-05	0.0075	T	0.01	0.20	0.9684	1.0000	T	0.06	0.08	0.4537	1.0000	T
p__Firmicutes; c__Clostridia; o__Clostridiales; f__Peptostreptococcaceae_[XI]; g__Peptostreptococcus; s__stomatis	-0.57	0.09	1.5E-08	1.1E-05	T	-0.19	0.20	0.3441	1.0000	T	-0.30	0.09	0.0008	0.4725	T
p__Fusobacteria; c__Fusobacteriia; o__Fusobacteriales; f__Fusobacteriaceae; g__Fusobacterium; s__nucleatum_subsp._polymorphum	0.87	0.10	8.9E-14	7.3E-11	T	0.74	0.21	0.0005	0.4003	T	0.11	0.09	0.1932	1.0000	T
p__Fusobacteria; c__Fusobacteriia; o__Fusobacteriales; f__Leptotrichiaceae; g__Leptotrichia	-0.59	0.09	4.5E-08	3.4E-05	T	0.45	0.21	0.0373	1.0000	T	-1.04	0.08	8.1E-17	6.6E-14	F
p__Fusobacteria; c__Fusobacteriia; o__Fusobacteriales; f__Leptotrichiaceae; g__Leptotrichia; s__sp._HMT_212	-0.47	0.11	4.7E-05	0.0316	T	0.59	0.20	0.0034	1.0000	T	0.12	0.10	0.2567	1.0000	T
Four taxa associated with eczema															
p__Firmicutes; c__Bacilli; o__Lactobacillales; f__Streptococcaceae; g__Streptococcus; s__gordonii	-0.17	0.09	0.0716	1.0000	T	-0.85	0.20	4.4E-05	0.0344	T	-0.41	0.09	8.1E-06	0.0056	F
p__Fusobacteria; c__Fusobacteriia; o__Fusobacteriales; f__Fusobacteriaceae; g__Fusobacterium; s__nucleatum_subsp._vincentii	0.22	0.10	0.0262	1.0000	T	1.24	0.21	4.3E-07	0.0003	T	-0.08	0.09	0.3555	1.0000	T
p__Fusobacteria; c__Fusobacteriia; o__Fusobacteriales; f__Fusobacteriaceae; g__Fusobacterium; s__sp._HMT_204	-0.06	0.10	0.5594	1.0000	T	1.15	0.21	2.3E-06	0.0018	T	0.11	0.08	0.1853	1.0000	T
p__Fusobacteria; c__Fusobacteriia; o__Fusobacteriales; f__Leptotrichiaceae; g__Leptotrichia; s__sp._HMT_212	0.59	0.13	1.4E-05	0.0098	F	1.11	0.24	5.7E-06	0.0045	T	0.61	0.12	7.0E-07	0.0005	F
Six taxa associated with rhinitis															
p__Bacteroidetes; c__Bacteroidia; o__Bacteroidales; f__Bacteroidales_[F-2]; g__Bacteroidales_[G-2]; s__bacterium_HMT_274	-0.53	0.12	2.7E-05	0.0185	F	-0.93	0.22	0.0001	0.0809	T	-0.80	0.11	4.9E-10	3.8E-07	T
p__Bacteroidetes; c__Bacteroidia; o__Bacteroidales; f__Porphyromonadaceae; g__Porphyromonas	-0.42	0.11	0.0003	0.1915	T	0.08	0.23	0.7238	1.0000	T	-0.57	0.10	3.2E-07	0.0002	T
p__Bacteroidetes; c__Flavobacteriia; o__Flavobacteriales; f__Flavobacteriaceae; g__Capnocytophaga; s__leadbetteri	-0.42	0.11	0.0001	0.0960	T	0.03	0.21	0.8940	1.0000	T	-0.68	0.10	1.8E-10	1.4E-07	T
p__Firmicutes; c__Clostridia; o__Clostridiales; f__Lachnospiraceae_[XIV]; g__Lachnoanaerobaculum; s__saburreum	0.27	0.09	0.0040	1.0000	T	1.00	0.19	5.7E-06	0.0045	F	0.46	0.08	6.1E-07	0.0004	T
p__Firmicutes; c__Clostridia; o__Clostridiales; f__Lachnospiraceae_[XIV]; g__Lachnospiraceae_[G-3]; s__bacterium_HMT_100	-0.37	0.09	0.0003	0.1676	T	-0.20	0.20	0.3284	1.0000	T	-0.61	0.08	1.6E-09	1.3E-06	T
p__Firmicutes; c__Negativicutes; o__Selenomonadales; f__Selenomonadaceae; g__Selenomonas; s__noxia	-0.16	0.09	0.0977	1.0000	T	-0.12	0.20	0.5486	1.0000	T	-0.42	0.08	7.2E-06	0.0050	T

This table summarizes results from the ANCOM-BC2 analysis determining taxa differentially abundant in relation to atopy, eczema, and rhinitis. Adjusted for age, sex, smoking (never/former/current), body mass index, and batch (1/2).
LFC, log fold change in natural log depicting differences in abundances relative to the reference group; SE, standard error; P, statistical significance; Q, statistical significance corrected for multiple testing; S, indicator denoting whether the taxon passed the sensitivity analysis (T for TRUE) or not (F for FALSE).

Table S4. Associations between asthma and the seventeen taxa differentially abundant in relation to atopy, eczema, or rhinitis (N=453).

	Asthma				
Taxon	LFC	SE	P	Q	S
Seven taxa associated with atopy					
p__Actinobacteria; c__Actinobacteria; o__Propionibacteriales; f__Propionibacteriaceae; g__Pseudopropionibacterium; s__propionicum	0.0002	0.39	0.9995	1.0000	T
p__Bacteroidetes; c__Bacteroidia; o__Bacteroidales; f__Prevotellaceae; g__Prevotella; s__sp._HMT_300	-0.08	0.40	0.8345	1.0000	T
p__Firmicutes	-0.31	0.39	0.4320	1.0000	T
p__Firmicutes; c__Clostridia; o__Clostridiales; f__Peptostreptococcaceae_[XI]; g__Peptostreptococcus; s__stomatis	-0.26	0.38	0.4995	1.0000	T
p__Fusobacteria; c__Fusobacteriia; o__Fusobacteriales; f__Fusobacteriaceae; g__Fusobacterium; s__nucleatum_subsp._polymorphum	0.70	0.39	0.0791	1.0000	T
p__Fusobacteria; c__Fusobacteriia; o__Fusobacteriales; f__Leptotrichiaceae; g__Leptotrichia	-0.40	0.40	0.3234	1.0000	T
p__Fusobacteria; c__Fusobacteriia; o__Fusobacteriales; f__Leptotrichiaceae; g__Leptotrichia; s__sp._HMT_212	-0.10	0.40	0.8014	1.0000	T
Four taxa associated with eczema					
p__Firmicutes; c__Bacilli; o__Lactobacillales; f__Streptococcaceae; g__Streptococcus; s__gordonii	-0.24	0.39	0.5439	1.0000	T
p__Fusobacteria; c__Fusobacteriia; o__Fusobacteriales; f__Fusobacteriaceae; g__Fusobacterium; s__nucleatum_subsp._vincentii	1.63	0.40	0.0002	0.1365	T
p__Fusobacteria; c__Fusobacteriia; o__Fusobacteriales; f__Fusobacteriaceae; g__Fusobacterium; s__sp._HMT_204	2.11	0.41	4.0E-06	0.0032	F
p__Fusobacteria; c__Fusobacteriia; o__Fusobacteriales; f__Leptotrichiaceae; g__Leptotrichia; s__sp._HMT_212	0.46	0.45	0.3051	1.0000	T
Six taxa associated with rhinitis					
p__Bacteroidetes; c__Bacteroidia; o__Bacteroidales; f__Bacteroidales_[F-2]; g__Bacteroidales_[G-2]; s__bacterium_HMT_274	-2.17	0.42	2.5E-06	0.0020	F
p__Bacteroidetes; c__Bacteroidia; o__Bacteroidales; f__Porphyromonadaceae; g__Porphyromonas	0.04	0.39	0.9168	1.0000	T
p__Bacteroidetes; c__Flavobacteriia; o__Flavobacteriales; f__Flavobacteriaceae; g__Capnocytophaga; s__leadbetteri	0.34	0.44	0.4475	1.0000	F
p__Firmicutes; c__Clostridia; o__Clostridiales; f__Lachnospiraceae_[XIV]; g__Lachnoanaerobaculum; s__saburreum	-0.77	0.39	0.0571	1.0000	T
p__Firmicutes; c__Clostridia; o__Clostridiales; f__Lachnospiraceae_[XIV]; g__Lachnospiraceae_[G-3]; s__bacterium_HMT_100	-0.98	0.42	0.0243	1.0000	T
p__Firmicutes; c__Negativicutes; o__Selenomonadales; f__Selenomonadaceae; g__Selenomonas; s__noxia	-1.38	0.39	0.0011	0.8153	T

This table summarizes results from the ANCOM-BC2 analysis determining taxa differentially abundant in relation to atopy, eczema, and rhinitis. Adjusted for age, sex, smoking (never/former/current), body mass index, and batch (1/2).

LFC, log fold change in natural log depicting differences in abundances relative to the reference group; SE, standard error; P, statistical significance; Q, statistical significance corrected for multiple testing; S, indicator denoting whether the taxon passed the sensitivity analysis (T for TRUE) or not (F for FALSE).

Table S5. Taxa differentially abundant in relation to a combined phenotype of atopy and asthma (N=453).

	Atopy without asthma					Asthma without atopy					Asthma with atopy				
Taxon	LFC	SE	P	Q	S	LFC	SE	P	Q	S	LFC	SE	P	Q	S
p__Bacteroidetes; c__Bacteroidia; o__Bacteroidales; f__Prevotellaceae; g__Prevotella	-0.53	0.16	0.0015	0.0202	T	0.63	0.91	0.4928	1.0000	T	0.09	0.65	0.8874	1.0000	T
p__Bacteroidetes; c__Bacteroidia; o__Bacteroidales; f__Prevotellaceae; g__Prevotella; s__sp._HMT_317	-0.94	0.16	2.9E-07	3.9E-06	T	0.29	0.89	0.7477	1.0000	T	1.11	0.64	0.0892	1.0000	T
p__Firmicutes; c__Clostridia; o__Clostridiales; f__Peptostreptococcaceae_[XI]; g__Peptostreptococcus; s__stomatis	-0.59	0.15	0.0002	0.0032	T	-0.02	0.88	0.9839	1.0000	T	-0.59	0.62	0.3422	1.0000	T
p__Firmicutes; c__Negativicutes; o__Veillonellales; f__Veillonellaceae; g__Veillonellaceae_[G-1]; s__bacterium_HMT_150	-0.55	0.17	0.0013	0.0178	T	-0.42	0.87	0.6325	1.0000	T	-0.14	0.61	0.8153	1.0000	T
p__Fusobacteria; c__Fusobacteriia; o__Fusobacteriales; f__Leptotrichiaceae; g__Leptotrichia	-0.51	0.16	0.0020	0.0263	T	0.48	0.90	0.5968	1.0000	T	-1.84	0.64	0.0060	0.0737	T
p__Fusobacteria; c__Fusobacteriia; o__Fusobacteriales; f__Leptotrichiaceae; g__Leptotrichia; s__sp._HMT_212	-0.51	0.16	0.0023	0.0305	T	-0.24	0.90	0.7931	1.0000	T	-0.30	0.61	0.6251	1.0000	T
p__Proteobacteria; c__Epsilonproteobacteria; o__Campylobacterales; f__Campylobacteraceae; g__Campylobacter; s__showae	-0.70	0.16	3.9E-05	0.0005	T	0.89	0.90	0.3259	1.0000	T	-0.07	0.66	0.9108	1.0000	T
p__Firmicutes; c__Bacilli; o__Lactobacillales; f__Streptococcaceae; g__Streptococcus	-0.13	0.18	0.4623	1.0000	T	-3.07	0.85	0.0004	0.0056	T	1.91	0.60	0.0018	0.0227	F
p__Firmicutes; c__Clostridia; o__Clostridiales; f__Lachnospiraceae_[XIV]; g__Catonella; s__morbi	-0.0007	0.16	0.9966	1.0000	T	-2.42	0.80	0.0029	0.0391	T	-0.17	0.62	0.7809	1.0000	T
p__Firmicutes; c__Negativicutes; o__Veillonellales; f__Veillonellaceae; g__Megasphaera; s__micronuciformis	-0.43	0.16	0.0104	0.1277	T	3.58	0.91	0.0002	0.0029	T	-1.54	0.64	0.0193	0.2182	T
p__Proteobacteria; c__Betaproteobacteria; o__Neisseriales; f__Neisseriaceae	1.97	0.16	5.5E-15	7.3E-14	F	0.65	0.91	0.4807	1.0000	T	2.06	0.66	0.0035	0.0433	T
p__Spirochaetes; c__Spirochaetia; o__Spirochaetales; f__Spirochaetaceae; g__Treponema; s__sp._HMT_270	0.27	0.16	0.1079	1.0000	T	0.26	0.85	0.7594	1.0000	T	2.12	0.60	0.0005	0.0064	T

This table summarizes results from the ANCOM-BC2 analysis determining taxa differentially abundant in relation to atopy, eczema, and rhinitis. Adjusted for age, sex, smoking (never/former/current), body mass index, and batch (1/2).

LFC, log fold change in natural log depicting differences in abundances relative to the reference group; SE, standard error; P, statistical significance; Q, statistical significance corrected for multiple testing; S, indicator denoting whether the taxon passed the sensitivity analysis (T for TRUE) or not (F for FALSE).

Table S6. Taxa differentially abundant in relation to a combined phenotype of atopy and eczema (N=453).

	Atopy without eczema					Eczema without atopy					Eczema with atopy				
Taxon	LFC	SE	P	Q	S	LFC	SE	P	Q	S	LFC	SE	P	Q	S
p__Bacteroidetes; c__Bacteroidia; o__Bacteroidales; f__Prevotellaceae; g__Prevotella	-0.68	0.16	0.0001	0.0010	T	-1.20	0.36	0.0020	0.0179	F	-0.51	0.49	0.3053	1	T
p__Firmicutes; c__Bacilli; o__Lactobacillales; f__Streptococcaceae; g__Streptococcus; s__mitis	-0.63	0.15	0.0002	0.0020	T	-0.78	0.37	0.0378	0.3417	T	-0.92	0.49	0.0654	0.5259	T
p__Firmicutes; c__Bacilli; o__Lactobacillales; f__Streptococcaceae; g__Streptococcus; s__sanguinis	-0.58	0.17	0.0008	0.0079	T	-0.20	0.36	0.5894	1	T	0.65	0.50	0.1932	1	T
p__Firmicutes; c__Clostridia; o__Clostridiales; f__Peptostreptococcaceae_[XI]; g__Peptostreptococcus; s__stomatis	-0.65	0.15	6.4E-05	0.0006	T	-0.31	0.36	0.4005	1	T	-1.75	0.47	0.0003	0.0032	T
p__Firmicutes; c__Clostridia; o__Clostridiales; f__Peptostreptococcaceae_[XI]; g__Peptostreptococcus; s__stomatis	-0.53	0.15	0.0010	0.0102	T	0.15	0.36	0.6715	1	T	-0.20	0.49	0.6792	1	T
p__Fusobacteria; c__Fusobacteriia; o__Fusobacteriales; f__Fusobacteriaceae; g__Fusobacterium; s__nucleatum_subsp._polymorphum	0.89	0.16	3.1E-07	3.1E-06	T	0.83	0.37	0.0272	0.2458	T	0.92	0.49	0.0620	0.4982	T
p__Fusobacteria; c__Fusobacteriia; o__Fusobacteriales; f__Leptotrichiaceae; g__Leptotrichia	-0.46	0.16	0.0047	0.0473	T	1.05	0.37	0.0065	0.0591	T	-0.53	0.49	0.2824	1	T
p__Fusobacteria; c__Fusobacteriia; o__Fusobacteriales; f__Leptotrichiaceae; g__Leptotrichia; s__sp._HMT_212	-0.54	0.17	0.0018	0.0182	T	0.38	0.35	0.2831	1	T	0.34	0.47	0.4631	1	F
p__Bacteroidetes; c__Bacteroidia; o__Bacteroidales; f__Prevotellaceae; g__Prevotella; s__micans	-0.68	0.16	0.0001	0.0007	F	-2.43	0.37	5.9E-09	5.9E-08	T	-0.80	0.51	0.1206	0.9693	T
p__Bacteroidetes; c__Bacteroidia; o__Bacteroidales; f__Prevotellaceae; g__Prevotella; s__buccae	0.14	0.16	0.3852	1.0000	T	-2.50	0.35	4.5E-10	4.5E-09	T	-1.78	0.49	0.0005	0.0047	F
p__Bacteroidetes; c__Flavobacteriia; o__Flavobacteriales; f__Flavobacteriaceae; g__Capnocytophaga	-0.17	0.16	0.2948	1.0000	F	-1.25	0.35	0.0007	0.0069	T	-0.47	0.48	0.3378	1	T
p__Fusobacteria; c__Fusobacteriia; o__Fusobacteriales; f__Leptotrichiaceae; g__Leptotrichia; s__sp._HMT_212	0.66	0.18	0.0003	0.0034	F	1.21	0.39	0.0026	0.0208	T	1.70	0.49	0.0008	0.0072	F
p__Bacteroidetes; c__Flavobacteriia; o__Flavobacteriales; f__Flavobacteriaceae; g__Capnocytophaga; s__sputigena	-0.04	0.17	0.8257	1.0000	T	0.11	0.35	0.7601	1	T	-1.97	0.47	5.5E-05	0.0006	T

This table summarizes results from the ANCOM-BC2 analysis determining taxa differentially abundant in relation to atopy, eczema, and rhinitis. Adjusted for age, sex, smoking (never/former/current), body mass index, and batch (1/2).

LFC, log fold change in natural log depicting differences in abundances relative to the reference group; SE, standard error; P, statistical significance; Q, statistical significance corrected for multiple testing; S, indicator denoting whether the taxon passed the sensitivity analysis (T for TRUE) or not (F for FALSE).

Table S7. Taxa differentially abundant in relation to a combined phenotype of atopy and rhinitis (N=453).

	Atopy without rhinitis					Rhinitis without atopy					Rhinitis with atopy				
Taxon	LFC	SE	P	Q	S	LFC	SE	P	Q	S	LFC	SE	P	Q	S
p__Firmicutes; c__Negativicutes; o__Veillonellales; f__Veillonellaceae; g__Veillonella	-0.78	0.24	0.0018	0.0159	T	-0.23	0.19	0.2475	1	T	0.07	0.21	0.7628	1	T
p__Firmicutes; c__Negativicutes; o__Veillonellales; f__Veillonellaceae; g__Veillonellaceae_[G-1]; s__bacterium_HMT_150	-0.76	0.25	0.0031	0.0269	T	0.17	0.21	0.4297	1	T	-0.30	0.23	0.1798	1	T
p__Fusobacteria; c__Fusobacteriia; o__Fusobacteriales; f__Fusobacteriaceae; g__Fusobacterium; s__nucleatum_subsp._polymorphum	1.76	0.24	2.7E-10	2.4E-09	T	-0.23	0.19	0.2409	1	F	0.58	0.22	0.0095	0.0726	F
p__Fusobacteria; c__Fusobacteriia; o__Fusobacteriales; f__Leptotrichiaceae; g__Leptotrichia; s__wadei	0.99	0.24	0.0001	0.0012	T	0.47	0.19	0.0185	0.1411	T	0.00	0.22	0.9942	1	F
p__Spirochaetes; c__Spirochaetia; o__Spirochaetales; f__Spirochaetaceae; g__Treponema; s__lecithinolyticum	-1.37	0.25	2.2E-06	1.9E-05	T	0.70	0.20	0.0009	0.0066	F	-0.59	0.22	0.0107	0.0711	T
k__Bacteria	-0.53	0.24	0.0304	0.2017	T	-0.63	0.19	0.0013	0.0112	T	-0.49	0.21	0.0227	0.1730	T
p__Bacteroidetes; c__Bacteroidia; o__Bacteroidales; f__Prevotellaceae; g__Prevotella; s__denticola	-0.68	0.24	0.0084	0.0644	F	0.63	0.20	0.0028	0.0245	T	-0.29	0.22	0.1881	1	T
p__Bacteroidetes; c__Bacteroidia; o__Bacteroidales; f__Prevotellaceae; g__Prevotella; s__oris	-0.40	0.27	0.1468	0.9738	T	-1.30	0.21	1.6E-08	0.0000	T	-0.43	0.24	0.0713	0.5439	T
p__Firmicutes; c__Bacilli; o__Lactobacillales; f__Streptococcaceae; g__Streptococcus; s__gordonii	-0.33	0.25	0.1814	1	T	-0.66	0.19	0.0010	0.0088	T	-0.39	0.21	0.0760	0.5801	T
p__Firmicutes; c__Bacilli; o__Lactobacillales; f__Streptococcaceae; g__Streptococcus; s__sanguinis	-1.62	0.25	2.8E-08	2.4E-07	F	-1.20	0.20	2.85E-07	0.0000	T	0.18	0.23	0.4218	1	F
p__Firmicutes; c__Bacilli; o__Lactobacillales; f__Streptococcaceae; g__Streptococcus; s__sanguinis	-0.39	0.24	0.1125	0.8589	T	-0.90	0.19	6.5E-06	0.0001	T	0.30	0.22	0.1675	1	T
p__Firmicutes; c__Clostridia; o__Clostridiales; f__Lachnospiraceae_[XIV]; g__Lachnospiraceae_[G-3]; s__bacterium_HMT_100	-0.30	0.25	0.2300	1	T	-0.63	0.19	0.0020	0.0156	T	-0.79	0.22	0.0006	0.0055	T
p__Bacteroidetes; c__Flavobacteriia; o__Flavobacteriales; f__Flavobacteriaceae; g__Capnocytophaga; s__sp._HMT_326	0.02	0.25	0.9522	1	T	0.11	0.20	0.5955	1	T	-0.68	0.21	0.0018	0.0157	T
p__Firmicutes; c__Bacilli; o__Lactobacillales; f__Streptococcaceae; g__Streptococcus; s__mitis	-0.59	0.24	0.0188	0.1438	T	-0.36	0.19	0.0720	0.4777	T	-0.75	0.21	0.0011	0.0092	T
p__Firmicutes; c__Clostridia; o__Clostridiales; f__Peptostreptococcaceae_[XI]; g__Peptostreptococcus; s__stomatis	-0.47	0.24	0.0601	0.3984	T	-0.38	0.19	0.0505	0.3858	T	-0.61	0.21	0.0052	0.0447	T
p__Fusobacteria; c__Fusobacteriia; o__Fusobacteriales; f__Leptotrichiaceae; g__Leptotrichia	0.03	0.24	0.8982	1	F	-0.88	0.19	4.1E-05	0.0003	F	-1.22	0.21	9.0E-07	7.8E-06	T
p__Proteobacteria; c__Epsilonproteobacteria; o__Campylobacterales; f__Campylobacteraceae; g__Campylobacter; s__showae	0.18	0.26	0.4760	1	T	-0.34	0.20	0.0938	0.7156	T	-0.78	0.22	0.0005	0.0044	T

This table summarizes results from the ANCOM-BC2 analysis determining taxa differentially abundant in relation to atopy, eczema, and rhinitis. Adjusted for age, sex, smoking (never/former/current), body mass index, and batch (1/2).

LFC, log fold change in natural log depicting differences in abundances relative to the reference group; SE, standard error; P, statistical significance; Q, statistical significance corrected for multiple testing; S, indicator denoting whether the taxon passed the sensitivity analysis (T for TRUE) or not (F for FALSE).

Table S8. Taxa differentially abundant in relation to nasal medication use among individuals with rhinitis (N=178).

	Nasal medication use in the past 12 months					Nasal medication use in the past week				
Taxon	LFC	SE	P	Q	S	LFC	SE	P	Q	S
p__Bacteroidetes; c__Bacteroidetes_[C-1]; o__Bacteroidetes_[O-1]; f__Bacteroidetes_[F-1]; g__Bacteroidetes_[G-3]; s__bacterium_HMT_365	-1.69	0.24	1.9E-05	0.0001	T	0.39	0.38	0.3270	1.0000	T
p__Bacteroidetes; c__Bacteroidia; o__Bacteroidales; f__Prevotellaceae; g__Prevotella	-1.68	0.24	1.6E-05	0.0001	T	-0.11	0.40	0.7868	1.0000	T
p__Bacteroidetes; c__Bacteroidia; o__Bacteroidales; f__Prevotellaceae; g__Prevotella; s__sp._HMT_475	1.11	0.24	0.0010	0.0064	T	1.67	0.39	0.0015	0.0082	F
p__Bacteroidetes; c__Flavobacteriia; o__Flavobacteriales; f__Flavobacteriaceae; g__Capnocytophaga	1.19	0.24	0.0004	0.0025	T	0.82	0.39	0.0580	0.3173	T
p__Firmicutes; c__Bacilli; o__Bacillales	-2.37	0.24	1.8E-07	1.1E-06	T	-2.25	0.38	5.4E-05	0.0003	F
p__Firmicutes; c__Bacilli; o__Lactobacillales; f__Streptococcaceae; g__Streptococcus; s__constellatus	-1.18	0.24	0.0001	0.0009	T	-0.95	0.40	0.0314	0.1718	T
p__Firmicutes; c__Clostridia; o__Clostridiales; f__Peptostreptococcaceae_[XI]; g__Peptostreptococcus; s__stomatis	-1.74	0.24	2.4E-05	0.0002	T	-1.01	0.39	0.0260	0.1421	T
p__Fusobacteria; c__Fusobacteriia; o__Fusobacteriales; f__Leptotrichiaceae; g__Leptotrichia; s__goodfellowii	-2.04	0.24	6.4E-07	4.2E-06	T	-1.70	0.40	0.0009	0.0048	F
p__Proteobacteria; c__Alphaproteobacteria; o__Rhodobacterales; f__Rhodobacteraceae	0.96	0.24	0.0033	0.0211	T	0.15	0.39	0.7057	1.0000	T
p__Proteobacteria; c__Betaproteobacteria; o__Neisseriales; f__Neisseriaceae; g__Eikenella; s__corrodens	-1.15	0.24	1.1E-05	6.8E-05	T	-0.62	0.38	0.1080	0.5904	T
p__Proteobacteria; c__Gammaproteobacteria; o__Pseudomonadales	-0.82	0.24	0.0041	0.0222	T	-1.54	0.38	0.0012	0.0080	F
p__Bacteroidetes; c__Bacteroidia; o__Bacteroidales; f__Prevotellaceae; g__Prevotella	-0.58	0.26	0.0314	0.1714	T	2.98	0.38	4.1E-08	2.7E-07	T
p__Bacteroidetes; c__Bacteroidia; o__Bacteroidales; f__Prevotellaceae; g__Prevotella; s__micans	-0.78	0.25	0.0055	0.0299	F	1.63	0.38	0.0004	0.0025	T
p__Firmicutes; c__Bacilli; o__Lactobacillales; f__Streptococcaceae; g__Streptococcus; s__anginosus	0.71	0.24	0.0168	0.0918	T	-2.35	0.39	0.0002	0.0012	T
p__Saccharibacteria_(TM7); c__Saccharibacteria_(TM7)_[C-1]; o__Saccharibacteria_(TM7)_[O-1]; f__Saccharibacteria_(TM7)_[F-1]; g__Saccharibacteria_(TM7)_[G-3]; s__bacterium_HMT_351	-0.50	0.24	0.0549	0.3001	T	-2.20	0.38	1.5E-05	9.8E-05	T

This table summarizes results from the ANCOM-BC2 analysis determining taxa differentially abundant in relation to atopy, eczema, and rhinitis. Adjusted for age, sex, smoking (never/former/current), body mass index, and batch (1/2). After excluding individuals with rhinitis without information on nasal medication use, 178 were used in the rhinitis and medication use analysis.

LFC, log fold change in natural log depicting differences in abundances relative to the reference group; SE, standard error; P, statistical significance; Q, statistical significance corrected for multiple testing; S, indicator denoting whether the taxon passed the sensitivity analysis (T for TRUE) or not (F for FALSE).

Table S9. Characteristics of study participants by sex.

	Males (n=240)	Females (n=213)
Age, years	29 ± 7	27 ± 7
Cigarette smoking status		
Never	161 (67%)	154 (72%)
Former	45 (19%)	29 (14%)
Current	34 (14%)	30 (14%)
Body mass index, kg/m^2	26 ± 4	24 ± 4
Current asthma		
No	229 (95%)	198 (93%)
Yes	11 (5%)	15 (7%)
Current atopy		
No	134 (56%)	150 (70%)
Yes	106 (44%)	63 (30%)
Current eczema		
No	217 (90%)	181 (85%)
Yes	23 (10%)	32 (15%)
Current rhinitis		
No	133 (55%)	117 (55%)
Yes	107 (45%)	96 (45%)
Current rhinitis - recent use of nasal medications [a]		
No medication use	53 (57%)	34 (40%)
Medication use in the past 12 months not past week	26 (28%)	33 (39%)
Medication use in last week	14 (15%)	18 (21%)
Current asthma combined with atopy		
Neither	131 (55%)	142 (67%)
Atopy without asthma	98 (41%)	56 (26%)
Asthma without atopy	3 (1%)	8 (4%)
Asthma with atopy	8 (3%)	7 (3%)
Current eczema combined with atopy		
Neither	121 (50%)	132 (62%)
Atopy without eczema	96 (40%)	49 (23%)
Eczema without atopy	13 (5%)	18 (8%)
Eczema with atopy	10 (4%)	14 (7%)
Current rhinitis combined with atopy		
Neither	91 (38%)	99 (46%)
Atopy without rhinitis	42 (17%)	18 (8%)
Rhinitis without atopy	43 (18%)	51 (24%)
Rhinitis with atopy	64 (27%)	45 (21%)
Batch		
2016	142 (59%)	127 (60%)
2019	98 (41%)	86 (40%)

N (%) or Mean ± Standard Deviation

[a] After excluding individuals with rhinitis without information on nasal medication use, 93 males 85 females were used in each sex-specific rhinitis and medication use analysis.

Table S10. Associations between overall bacterial diversity and allergy outcomes stratified by sex.

	Richness			Shannon Index			Faith's Phylogenetic Diversity		
	Coef	SE	P	Coef	SE	P	Coef	SE	P
**Participants: males only (n=240)**									
Asthma	23.73	17.88	0.1858	0.21	0.12	0.0833	1.20	0.92	0.1897
Atopy	-11.85	7.59	0.1198	-0.10	0.05	0.0595	-0.17	0.39	0.6557
Eczema	6.04	12.84	0.6386	-0.04	0.09	0.6381	-0.16	0.66	0.8052
Rhinitis	-18.66	7.48	0.0133	-0.10	0.05	0.0399	-1.35	0.38	0.0004
Asthma and atopy combined									
Atopy without asthma	-14.27	7.77	0.0675	-0.11	0.05	0.0330	-0.30	0.40	0.4578
Asthma without atopy	7.85	33.78	0.8164	0.18	0.23	0.4134	0.15	1.74	0.9311
Asthma with atopy	21.10	21.14	0.3193	0.15	0.14	0.2907	1.42	1.09	0.1920
Atopy and eczema combined									
Atopy without eczema	-12.69	8.01	0.1148	-0.08	0.05	0.1370	-0.29	0.41	0.4830
Eczema without atopy	1.73	17.23	0.9200	0.03	0.12	0.7707	-0.69	0.88	0.4368
Eczema with atopy	-1.68	19.38	0.9312	-0.22	0.13	0.0900	0.22	0.99	0.8275
Atopy and rhinitis combined									
Atopy without rhinitis	14.90	10.60	0.1612	0.08	0.07	0.2828	0.58	0.55	0.2904
Rhinitis without atopy	5.34	10.54	0.6129	0.06	0.07	0.3631	-1.04	0.54	0.0566
Rhinitis with atopy	-27.31	9.31	0.0037	-0.18	0.06	0.0044	-1.25	0.48	0.0099
Nasal medication use in individuals with rhinitis [a]									
Medication use in the past 12 months not past week	-3.61	13.18	0.7845	-0.17	0.09	0.0747	0.04	0.63	0.9511
Medication use in last week	-13.29	16.64	0.4266	-0.14	0.12	0.2433	-0.22	0.80	0.7842
**Participants: females only (n=213)**									
Asthma	-13.72	15.87	0.3883	-0.06	0.11	0.6138	-0.49	0.78	0.5294
Atopy	-11.09	8.79	0.2085	-0.08	0.06	0.1811	-0.57	0.43	0.1867
Eczema	4.41	11.26	0.6955	0.03	0.08	0.7409	0.15	0.55	0.7821
Rhinitis	-7.89	8.01	0.3257	-0.02	0.06	0.6937	-0.42	0.39	0.2900
Asthma and atopy combined									
Atopy without asthma	-14.10	9.26	0.1291	-0.10	0.06	0.1127	-0.70	0.46	0.1243
Asthma without atopy	-31.70	21.32	0.1387	-0.16	0.15	0.2804	-1.20	1.05	0.2557
Asthma with atopy	-0.14	22.59	0.9952	0.01	0.16	0.9306	-0.03	1.11	0.9782
Atopy and eczema combined									
Atopy without eczema	-14.42	9.82	0.1435	-0.08	0.07	0.2440	-0.72	0.48	0.1399
Eczema without atopy	0.28	14.72	0.9849	0.05	0.10	0.5988	-0.01	0.72	0.9849
Eczema with atopy	0.63	16.40	0.9693	-0.06	0.11	0.6002	-0.08	0.81	0.9183
Atopy and rhinitis combined									
Atopy without rhinitis	8.90	14.77	0.5476	0.02	0.10	0.8762	0.70	0.72	0.3365
Rhinitis without atopy	3.68	10.12	0.7164	0.05	0.07	0.4585	0.29	0.50	0.5595
Rhinitis with atopy	-17.64	10.46	0.0932	-0.10	0.07	0.1786	-0.96	0.51	0.0614
Nasal medication use in individuals with rhinitis [a]									
Medication use in the past 12 months not past week	-14.29	15.00	0.3435	-0.07	0.11	0.5393	-1.20	0.73	0.1040
Medication use in last week	-16.62	18.04	0.3596	-0.05	0.13	0.7286	-1.18	0.88	0.1812

Adjusted for age, smoking (never/former/current), body mass index, and batch (1/2).

[a] After excluding individuals with rhinitis without information on nasal medication use, 93 males 85 females were used in each sex-specific rhinitis and medication use analysis.

Table S11. Bacterial taxa differentially abundant in relation to atopy, eczema, and rhinitis stratified by sex.

	Atopy					Eczema					Rhinitis				
Taxon	LFC	SE	P	Q	S	LFC	SE	P	Q	S	LFC	SE	P	Q	S
**Participants: males only (n=240)**															
11 taxa associated with atopy															
p__Bacteroidetes; c__Bacteroidia; o__Bacteroidales; f__Prevotellaceae; g__Prevotella	-1.19	0.13	7.90E-08	6.61E-05	T	-0.86	0.35	0.0233	1	T	0.44	0.13	0.0037	1	T
p__Bacteroidetes; c__Bacteroidia; o__Bacteroidales; f__Prevotellaceae; g__Prevotella; s__intermedia	-1.38	0.16	4.91E-08	4.12E-05	T	4.85	0.36	4.43E-11	3.77E-08	F	0.98	0.16	4.98E-06	0.0040	F
p__Bacteroidetes; c__Bacteroidia; o__Bacteroidales; f__Prevotellaceae; g__Prevotella; s__oris	-1.05	0.14	1.18E-07	9.82E-05	T	0.17	0.37	0.6372	1	T	0.00	0.14	0.9969	1	T
p__Bacteroidetes; c__Bacteroidia; o__Bacteroidales; f__Prevotellaceae; g__Prevotella; s__sp._HMT_300	-0.83	0.15	3.89E-06	0.003	T	1.44	0.35	0.0003	0.2448	T	0.48	0.14	0.0024	1	T
p__Firmicutes; c__Clostridia; o__Clostridiales; f__Lachnospiraceae_[XIV]; g__Catonella; s__morbi	-0.88	0.14	4.69E-07	3.87E-04	T	0.93	0.36	0.0132	1	T	0.40	0.14	0.0072	1	T
p__Firmicutes; c__Clostridia; o__Clostridiales; f__Peptostreptococcaceae_[XI]; g__Peptostreptococcaceae_[XI][G-7]; s__yurii_subspp._yurii_&_margaretiae	-0.69	0.14	1.30E-05	0.010	T	0.41	0.35	0.2499	1	F	0.37	0.14	0.0101	1	T
p__Firmicutes; c__Clostridia; o__Clostridiales; f__Peptostreptococcaceae_[XI]; g__Peptostreptococcus; s__stomatis	-0.76	0.13	4.19E-06	0.003	T	0.18	0.35	0.6188	1	F	-0.57	0.13	0.0001	0.1008	T
p__Firmicutes; c__Negativicutes; o__Veillonellales; f__Veillonellaceae; g__Veillonellaceae_[G-1]; s__bacterium_HMT_150	-0.78	0.17	1.42E-05	0.011	T	-0.20	0.37	0.5861	1	T	-0.42	0.16	0.0125	1	T
p__Fusobacteria; c__Fusobacteriia; o__Fusobacteriales; f__Leptotrichiaceae; g__Leptotrichia; s__hofstadii	-1.59	0.14	5.10E-10	4.36E-07	T	0.05	0.38	0.8923	1	T	0.41	0.14	0.0072	1	F
p__Saccharibacteria_(TM7); c__Saccharibacteria_(TM7)_[C-1]; o__Saccharibacteria_(TM7)_[O-1]; f__Saccharibacteria_(TM7)_[F-1]; g__Saccharibacteria_(TM7)_[G-1]; s__bacterium_HMT_349	-1.22	0.15	2.07E-10	1.77E-07	T	1.17	0.34	0.0012	0.9399	T	0.44	0.15	0.0062	1	F
p__Spirochaetes; c__Spirochaetia; o__Spirochaetales; f__Spirochaetaceae; g__Treponema; s__lecithinolyticum	-1.30	0.15	5.15E-09	4.38E-06	T	-0.09	0.36	0.8045	1	T	-0.32	0.14	0.0320	1	T
Two taxa associated with eczema															
p__Fusobacteria; c__Fusobacteriia; o__Fusobacteriales; f__Fusobacteriaceae; g__Fusobacterium; s__sp._HMT_204	-0.07	0.14	0.6115	1	T	1.90	0.36	2.32E-05	0.0191	T	-0.42	0.13	0.0040	1	T
p__Fusobacteria; c__Fusobacteriia; o__Fusobacteriales; f__Fusobacteriaceae; g__Fusobacterium	0.30	0.14	0.0412	1	T	1.89	0.36	4.86E-05	0.0400	T	-0.07	0.13	0.5850	1	T
Three taxa associated with rhinitis															
p__Actinobacteria; c__Actinobacteria; o__Actinomycetales; f__Actinomycetaceae; g__Actinomyces; s__sp._HMT_171	0.13	0.13	0.3487	1	T	0.00	0.36	0.9994	1	T	-0.87	0.13	1.64E-06	0.0013	T
p__Actinobacteria; c__Actinobacteria; o__Corynebacteriales; f__Corynebacteriaceae; g__Corynebacterium; s__durum	-0.44	0.14	0.0045	1	T	0.00	0.37	0.9918	1	F	-0.88	0.13	1.45E-06	0.0012	T
p__Firmicutes; c__Clostridia; o__Clostridiales; f__Lachnospiraceae_[XIV]; g__Lachnospiraceae_[G-3]; s__bacterium_HMT_100	-0.12	0.13	0.3823	1	T	-0.12	0.35	0.7361	1	T	-1.00	0.13	1.70E-07	0.0001	T

**Participants: females only (n=213)**															
Seven taxa associated with atopy															
p__Bacteroidetes; c__Bacteroidia; o__Bacteroidales; f__Bacteroidales_[F-2]; g__Bacteroidales_[G-2]; s__bacterium_HMT_274	0.94	0.15	8.83E-06	0.0063	T	-0.11	0.22	0.6377	1	T	0.30	0.10	0.0066	1	T
p__Bacteroidetes; c__Flavobacteriia; o__Flavobacteriales; f__Flavobacteriaceae; g__Capnocytophaga; s__gingivalis	-0.77	0.12	2.32E-06	0.0017	T	0.71	0.21	0.0025	1	T	-0.20	0.08	0.0254	1	T
p__Bacteroidetes; c__Flavobacteriia; o__Flavobacteriales; f__Flavobacteriaceae; g__Capnocytophaga; s__leadbetteri	1.32	0.17	3.86E-09	3.02E-06	T	0.31	0.26	0.2357	1	T	0.78	0.13	4.92E-07	0.0003	F
p__Bacteroidetes; c__Flavobacteriia; o__Flavobacteriales; f__Flavobacteriaceae; g__Capnocytophaga; s__sp._HMT_336	-1.54	0.12	2.47E-11	1.98E-08	T	0.75	0.20	0.0012	0.7853	T	0.40	0.08	0.0001	0.0618	T
p__Firmicutes	0.55	0.12	6.66E-05	0.0439	T	0.22	0.20	0.2722	1	T	-0.11	0.08	0.1743	1	T
p__Fusobacteria; c__Fusobacteriia; o__Fusobacteriales; f__Leptotrichiaceae; g__Leptotrichia; s__sp._HMT_225	-0.75	0.13	4.91E-06	0.0035	T	0.01	0.21	0.9565	1	T	0.25	0.10	0.0158	1	T
p__Spirochaetes; c__Spirochaetia; o__Spirochaetales; f__Spirochaetaceae; g__Treponema	0.82	0.15	8.05E-06	0.0057	T	0.67	0.26	0.0137	1	T	0.76	0.11	1.21E-07	8.53E-05	F
Two taxa associated with eczema															
p__Bacteroidetes; c__Flavobacteriia; o__Flavobacteriales; f__Flavobacteriaceae; g__Capnocytophaga; s__sputigena	0.50	0.12	0.0007	0.4162	T	1.36	0.21	5.75E-06	0.0043	T	0.71	0.08	6.60E-08	4.69E-05	F
p__Fusobacteria; c__Fusobacteriia; o__Fusobacteriales; f__Fusobacteriaceae; g__Fusobacterium	-0.05	0.13	0.6724	1	T	1.04	0.20	1.18E-05	0.0087	T	-0.04	0.08	0.6153	1	T
Eight taxa associated with rhinitis															
p__Bacteroidetes; c__Bacteroidia; o__Bacteroidales; f__Porphyromonadaceae; g__Porphyromonas	0.91	0.13	4.39E-07	0.0003	F	-1.65	0.20	5.68E-08	4.50E-05	F	-0.56	0.09	2.01E-06	0.0013	T
p__Bacteroidetes; c__Flavobacteriia; o__Flavobacteriales; f__Flavobacteriaceae; g__Bergeyella; s__sp._HMT_322	-0.96	0.15	2.42E-06	0.0018	F	-0.37	0.20	0.0890	1	T	-0.59	0.10	1.52E-05	0.0094	T
p__Bacteroidetes; c__Flavobacteriia; o__Flavobacteriales; f__Flavobacteriaceae; g__Capnocytophaga; s__leadbetteri	-0.59	0.16	0.0006	0.3827	T	0.74	0.23	0.0029	1	T	-0.93	0.13	2.09E-08	1.52E-05	T
p__Firmicutes; c__Bacilli; o__Lactobacillales; f__Streptococcaceae; g__Streptococcus	-0.84	0.18	2.17E-05	0.0149	F	-0.20	0.30	0.5111	1	T	-0.66	0.15	4.26E-05	0.0253	T
p__Firmicutes; c__Bacilli; o__Lactobacillales; f__Streptococcaceae; g__Streptococcus; s__sanguinis	-0.94	0.15	1.02E-06	0.0008	F	0.53	0.25	0.0419	1	T	-0.57	0.11	1.50E-05	0.0093	T
p__Firmicutes; c__Negativicutes; o__Veillonellales; f__Veillonellaceae; g__Veillonella; s__sp._HMT_780	-0.51	0.12	0.0003	0.1592	T	-0.35	0.20	0.0932	1	T	-0.40	0.08	2.01E-05	0.0123	T
p__Fusobacteria; c__Fusobacteriia; o__Fusobacteriales; f__Fusobacteriaceae; g__Fusobacterium; s__sp._HMT_203	0.14	0.15	0.3418	1	T	0.72	0.21	0.0022	1	T	0.86	0.10	1.24E-08	9.09E-06	T
p__Fusobacteria; c__Fusobacteriia; o__Fusobacteriales; f__Leptotrichiaceae; g__Leptotrichia	-0.46	0.13	0.0017	0.9865	T	1.08	0.23	0.0001	0.0772	T	-0.88	0.09	3.99E-09	2.98E-06	T

This table summarizes results from the ANCOM-BC2 analysis determining taxa differentially abundant in relation to atopy, eczema, and rhinitis. Adjusted for age, smoking (never/former/current), body mass index, and batch (1/2).

LFC, log fold change in natural log depicting differences in abundances relative to the reference group; SE, standard error; P, statistical significance; Q, statistical significance corrected for multiple testing; S, indicator denoting whether the taxon passed the sensitivity analysis (T for TRUE) or not (F for FALSE).

Table S12. Taxa differentially abundant in relation to a combined phenotype of atopy and rhinitis stratified by sex.

	Atopy without rhinitis					Rhinitis without atopy					Rhinitis with atopy				
Taxon	LFC	SE	P	Q	S	LFC	SE	P	Q	S	LFC	SE	P	Q	S
**Participants: males only (n=240)**															
p__Bacteroidetes; c__Bacteroidia; o__Bacteroidales; f__Prevotellaceae; g__Prevotella; s__oris	-1.22	0.35	0.0008	0.0081	T	-1.47	0.34	6.00E-05	0.0007	T	-0.36	0.35	0.3086	1	T
p__Bacteroidetes; c__Bacteroidia; o__Bacteroidales; f__Prevotellaceae; g__Prevotella; s__sp._HMT_317	-5.82	0.32	1.51E-12	1.77E-11	T	-0.02	0.34	0.9544	1	T	0.08	0.34	0.8088	1	T
p__Firmicutes; c__Clostridia; o__Clostridiales; f__Peptostreptococcaceae_[XI]; g__Peptostreptococcus; s__stomatis	-1.33	0.32	0.0003	0.0040	T	-0.93	0.31	0.0062	0.0659	T	-0.76	0.33	0.0284	0.2754	F
p__Firmicutes; c__Negativicutes; o__Veillonellales; f__Veillonellaceae; g__Veillonellaceae_[G-1]; s__bacterium_HMT_150	-1.17	0.33	0.0007	0.0083	T	-0.50	0.33	0.1299	1	T	-0.70	0.34	0.0412	0.4410	T
p__Fusobacteria; c__Fusobacteriia; o__Fusobacteriales; f__Fusobacteriaceae; g__Fusobacterium; s__nucleatum_subsp._polymorphum	1.30	0.32	0.0003	0.0033	T	-0.15	0.31	0.6195	1	F	0.93	0.33	0.0080	0.0856	T
p__Fusobacteria; c__Fusobacteriia; o__Fusobacteriales; f__Leptotrichiaceae; g__Leptotrichia; s__sp._HMT_879	-1.43	0.32	0.0001	0.0012	T	-0.34	0.32	0.2896	1	T	1.42	0.34	0.0002	0.0023	F
p__Fusobacteria; c__Fusobacteriia; o__Fusobacteriales; f__Leptotrichiaceae; g__Leptotrichia; s__wadei	1.45	0.32	7.15E-05	0.0008	T	0.91	0.32	0.0071	0.0759	T	0.13	0.34	0.6958	1	T
p__Spirochaetes; c__Spirochaetia; o__Spirochaetales; f__Spirochaetaceae; g__Treponema; s__lecithinolyticum	-2.17	0.33	1.38E-06	1.61E-05	T	-0.02	0.32	0.9599	1	T	-0.99	0.34	0.0078	0.0830	T
p__Actinobacteria; c__Actinobacteria; o__Actinomycetales; f__Actinomycetaceae; g__Actinomyces; s__sp._HMT_171	0.06	0.32	0.8601	1	T	-1.57	0.31	8.27E-05	0.0010	T	-0.43	0.33	0.2084	1	T
p__Firmicutes; c__Bacilli; o__Lactobacillales; f__Streptococcaceae; g__Streptococcus	-0.87	0.38	0.0253	0.2710	T	1.35	0.34	0.0003	0.0036	T	0.73	0.34	0.0394	0.3826	T
p__Firmicutes; c__Clostridia; o__Clostridiales; f__Lachnospiraceae_[XIV]; g__Lachnospiraceae_[G-3]; s__bacterium_HMT_100	0.31	0.32	0.3519	1	T	-1.43	0.31	0.0002	0.0024	T	-0.71	0.33	0.0457	0.4889	T
p__Fusobacteria; c__Fusobacteriia; o__Fusobacteriales; f__Leptotrichiaceae; g__Leptotrichia; s__hofstadii	-1.54	0.33	0.0002	0.0024	F	0.51	0.31	0.1212	1	F	-1.24	0.33	0.0017	0.0183	T
**Participants: females only (n=213)**															
p__Bacteroidetes; c__Bacteroidia; o__Bacteroidales; f__Bacteroidales_[F-2]; g__Bacteroidales_[G-2]; s__bacterium_HMT_274	1.99	0.46	0.0007	0.0044	T	1.26	0.22	6.23E-05	0.0004	F	1.01	0.29	0.0033	0.0165	F
p__Bacteroidetes; c__Bacteroidia; o__Bacteroidales; f__Porphyromonadaceae; g__Porphyromonas	2.57	0.47	2.99E-05	0.0002	T	1.97	0.24	1.30E-07	9.06E-07	F	1.42	0.28	7.79E-05	0.0004	F
p__Bacteroidetes; c__Bacteroidia; o__Bacteroidales; f__Prevotellaceae; g__Prevotella; s__pleuritidis	1.73	0.44	0.0004	0.0031	T	0.28	0.30	0.3498	1	T	-1.09	0.29	0.0007	0.0044	F
p__Firmicutes; c__Bacilli; o__Lactobacillales; f__Streptococcaceae; g__Streptococcus; s__cristatus_clade_578	1.47	0.44	0.0041	0.0285	T	0.22	0.22	0.3399	1	T	0.79	0.27	0.0096	0.0575	T
p__Proteobacteria; c__Gammaproteobacteria; o__Pasteurellales; f__Pasteurellaceae; g__Aggregatibacter	1.43	0.46	0.0048	0.0288	T	0.36	0.23	0.1403	0.6993	T	-1.06	0.28	0.0008	0.0055	F
p__Bacteroidetes; c__Flavobacteriia; o__Flavobacteriales; f__Flavobacteriaceae; g__Capnocytophaga; s__leadbetteri	-0.55	0.42	0.1981	1	T	-1.54	0.23	4.65E-07	3.24E-06	T	-0.28	0.28	0.3294	1	T
p__Bacteroidetes; c__Bacteroidia; o__Bacteroidales; f__Prevotellaceae; g__Alloprevotella; s__tannerae	-1.32	0.42	0.0033	0.0198	F	-1.30	0.26	1.46E-05	0.0001	T	0.62	0.30	0.0467	0.2325	T
p__Bacteroidetes; c__Bacteroidia; o__Bacteroidales; f__Prevotellaceae; g__Prevotella	-1.06	0.42	0.0217	0.1295	T	-0.70	0.22	0.0058	0.0406	T	0.55	0.28	0.0609	0.3036	T

p__Bacteroidetes; c__Flavobacteriia; o__Flavobacteriales; f__Flavobacteriaceae; g__Capnocytophaga; s__leadbetteri	-0.14	0.44	0.7474	1	T	-0.83	0.26	0.0027	0.0162	T	-1.07	0.30	0.0009	0.0065	T
p__Firmicutes; c__Bacilli; o__Lactobacillales; f__Streptococcaceae; g__Streptococcus	-0.09	0.42	0.8261	1	T	-1.25	0.23	5.02E-05	0.0004	T	-1.04	0.27	0.0015	0.0091	F
p__Firmicutes; c__Clostridia; o__Clostridiales; f__Lachnospiraceae_[XIV]; g__Lachnoanaerobaculum; s__umeaense	-1.49	0.42	0.0018	0.0090	F	-1.71	0.23	1.67E-07	9.99E-07	T	-2.61	0.28	3.28E-09	2.29E-08	F
p__Fusobacteria; c__Fusobacteriia; o__Fusobacteriales; f__Leptotrichiaceae; g__Leptotrichia	-0.18	0.43	0.6847	1	T	-0.89	0.23	0.0009	0.0062	T	-1.08	0.28	0.0009	0.0062	T
p__Fusobacteria; c__Fusobacteriia; o__Fusobacteriales; f__Leptotrichiaceae; g__Leptotrichia; s__sp._HMT_498	-0.97	0.43	0.0361	0.1797	T	1.35	0.23	1.36E-05	8.14E-05	T	1.86	0.28	2.77E-06	1.94E-05	F
p__Saccharibacteria_(TM7); c__Saccharibacteria_(TM7)_[C-1]; o__Saccharibacteria_(TM7)_[O-1]; f__Saccharibacteria_(TM7)_[F-1]; g__Saccharibacteria_(TM7)_[G-1]; s__bacterium_HMT_346	-0.98	0.47	0.0400	0.1992	T	-0.82	0.25	0.0015	0.0104	T	-0.76	0.33	0.0226	0.1354	T
p__Firmicutes; c__Bacilli; o__Lactobacillales; f__Streptococcaceae; g__Streptococcus	-0.53	0.42	0.2169	1	T	0.24	0.25	0.3343	1	T	1.34	0.28	3.20E-05	0.0002	T
p__Proteobacteria; c__Epsilonproteobacteria; o__Campylobacterales; f__Campylobacteraceae; g__Campylobacter; s__showae	1.18	0.43	0.0102	0.0610	T	-0.13	0.23	0.5611	1	T	-1.44	0.28	1.61E-05	0.0001	T
p__Proteobacteria; c__Gammaproteobacteria; o__Pasteurellales; f__Pasteurellaceae; g__Haemophilus; s__parainfluenzae	-0.70	0.47	0.1396	0.6956	T	-0.63	0.23	0.0101	0.0602	T	-1.11	0.27	0.0002	0.0011	T

This table summarizes results from the ANCOM-BC2 analysis determining taxa differentially abundant in relation to atopy, eczema, and rhinitis. Adjusted for age, smoking (never/former/current), body mass index, and batch (1/2).

LFC, log fold change in natural log depicting differences in abundances relative to the reference group; SE, standard error; P, statistical significance; Q, statistical significance corrected for multiple testing; S, indicator denoting whether the taxon passed the sensitivity analysis (T for TRUE) or not (F for FALSE).

Table S13. Associations between overall bacterial diversity and use of different nasal medication types in individuals with rhinitis.

		Richness			Shannon Index			Faith's Phylogenetic Diversity		
	N	Coef	SE	P	Coef	SE	P	Coef	SE	P
Medication use in the past 12 months (not past week)										
Antihistamines only	32	-15.50	12.39	0.2130	-0.16	0.09	0.0737	-0.85	0.59	0.1486
Steroids only	7	-9.20	23.41	0.6949	-0.18	0.17	0.2754	-0.11	1.11	0.9227
Both	20	0.68	14.93	0.9639	-0.03	0.11	0.7480	-0.31	0.71	0.6616
Medication use in last week										
Antihistamines only	22	-11.50	13.61	0.3999	-0.07	0.10	0.5025	0.10	0.64	0.8718
Steroids only	6	0.53	24.75	0.9830	-0.07	0.18	0.6829	-0.93	1.17	0.4247
Both	4	-54.10	28.94	0.0643	-0.27	0.21	0.2093	-4.16	1.36	0.0029

Adjusted for age, sex, smoking (never/former/current), body mass index, and batch (1/2).
Referent group: 87 individuals with rhinitis reporting no use of medication use.
